# Antifungal activity of oily core PEGylated PLGA nanocapsules loaded with *Penicillium oxalicum* fungal extract: in vitro, in vivo, and in silico study

**DOI:** 10.1186/s12934-025-02891-x

**Published:** 2026-01-16

**Authors:** Engy Elekhnawy, Dalia H. Abdelkader, Duaa Eliwa, Sarah Ibrahim, Moataz A. Shaldam, Walaa A. Negm

**Affiliations:** 1 Microbiology and Immunology Department, Faculty of Pharmacy, Tanta University, Tanta, 31527 Egypt; 2 Department of Pharmaceutical Technology, Faculty of Pharmacy, Tanta University, Tanta, 31527 Egypt; 3 Department of Pharmacognosy, Faculty of Pharmacy, Tanta University, Tanta, 31527 Egypt; 4 Human Anatomy and Embryology Department, Faculty of Medicine, Tanta University, Tanta, 31527 Egypt; 5 Department of Pharmaceutical Chemistry, Faculty of Pharmacy, Kafrelsheikh University, Kafr El-Sheikh, 33516 Egypt

**Keywords:** *Penicillium oxalicum*, Endophytic fungi, LC-HRMS/MS, Hybrid single emulsion/nanoprecipitation, Animal model

## Abstract

The rise of difficult-to-treat fungal infections necessitates novel therapeutic strategies. In this study, endophytic fungi were isolated from *Acalypha hispida* leaves and molecularly identified as *Penicillium oxalicum* via 18S rRNA sequencing. LC–MS/MS analysis of the fungal extract revealed major bioactive compounds, including linoleic acid, sinapinic acid, alternariol monomethyl ether, ellagic acid, and kaurenic acid. Oily-core poly (ethylene glycol) methyl ether-block-poly(lactide-*co*-glycolide) nanocapsules (PEGylated PLGA NCs) were developed to encapsulate the fungal extract, improving stability and bioavailability. The PEGylated PLGA NCs exhibited controlled particle size, positive surface charge, and spherical morphology. In vitro, the PEGylated PLGA NCs demonstrated antifungal activity against *Candida albicans* with inhibition zones of 10–14 mm. In vivo, treatment significantly improved histological features of the kidney, liver, and spleen, and reduced tumor necrosis factor-alpha and cyclooxygenase-2 expression. In silico studies further confirmed the potential of the major compounds of the fungal extract to inhibit *C. albicans* aspartic proteinases SAP4-6. These findings suggest that PEGylated PLGA NCs loaded with *P. oxalicum* extract represent a promising antifungal therapeutic strategy.

**Supplementary Information:**

The online version contains supplementary material available at 10.1186/s12934-025-02891-x.

## Introduction

The rising incidence of fungal infections, along with the emergence of resistant strains, poses a significant global challenge in clinical practice. *Candida albicans*, a commensal member of the human microbiota, can shift to a pathogenic form under certain conditions, particularly in immunocompromised individuals. Numerous fungal pathogens are capable of causing a wide spectrum of infections, ranging from superficial to severe, life-threatening systemic infections [1].

Though effective antifungal drugs are present, they are often linked with many side effects. This is in addition to the large escalation in the percentage of resistant fungal isolates [2]. Thus, there is a great necessity to explore new substitute therapeutic modalities for the conventional antifungal drugs. Recently, natural sources, like plants and microorganisms, have gained much attention as they are rich with many pharmacologically active constituents [3].

Endophytic bacteria and fungi are present in the plant tissues without triggering any harm to their host. Fungal endophytes produce diverse metabolites, including steroids, alkaloids, flavonoids, terpenoids, quinones, glycosides, isocoumarins, xanthones, lactones, phenyl propanoids, and lignans, with potential therapeutic applications [4].

In parallel, polymeric nanocapsules (NCs) have emerged as versatile drug delivery systems due to their biocompatibility, enhanced stability, improved cellular uptake, and controlled release properties. The preparation technique should be carefully selected to manage the physical properties of the prepared NCs, regarding size distribution, particle size, and surface charge. The particle size is a crucial feature that should be tightly controlled to be in the optimum range, not too small to overcome rapid penetration into blood vessels, and also not too big to bypass the immune system elimination [5].

Among polymeric systems, poly (ethylene glycol) methyl ether-block-poly(lactide-*co*-glycolide) nanocapsules (PEGylated PLGA NCs) is widely used, as its amphiphilic nature allows self-assembly into NCs in aqueous media [6]. Additionally, it provides stealth properties (reduced opsonization) to PLGA NPs, enabling extended circulation [7, 8].

The aim of this study was to isolate endophytic fungi from *Acalypha hispida* leaves, identify their major bioactive compounds, and formulate PEGylated PLGA NCs loaded with the fungal extract using a hybrid single o/w emulsion-nanoprecipitation method, in order to evaluate their antifungal potential in vitro, in vivo, and in silico.

## Materials and methods

### Chemicals and plant

Poly (ethylene glycol) methyl ether-block-poly(lactide-*co*-glycolide) PEG average Mn 5000, PLGA average Mn 25,000, lactide: glycolide 50:50 (PEG-PLGA, Catalogue No. 799041) was purchased from Sigma-Aldrich Chemical Co. (St. Louis, MO, USA). Span 80 was purchased from Oxford Lab Fine Chem LLP (Navghar, India). Piochem for Laboratory Chemicals (October City, Egypt) manufactured and packed oleic acid. Acetone, dimethylformamide (DMF), dichloromethane (DCM), and propylene glycol were supplied from Al-Gomhoria Company (Cairo, Egypt). All media were obtained from Oxoid (Basingstoke, UK).

### Plant

Fresh and healthy leaves of *Acalypha hispida* Burm. f. were obtained from a local farm in Tanta City, Al-Gharbia Governorate, Egypt. The plant was taxonomically identified by Dr. Esraa Ammar from the Faculty of Science, Tanta University. A voucher specimen (PG-A-D-133) was deposited at the Pharmacognosy Department, Tanta University.

### Isolation and purification of the endophytic fungus *Penicillium oxalicum* from *Acalypha hispida* leaves

Fresh *A. hispida* leaves were initially washed with tap water, then surface-sterilized by immersing in 70% ethanol for one minute, followed by rinsing three to four times with sterile distilled water, and subsequently air-dried. The sterilized leaves were then cut into 2 × 2 cm sections and placed on potato dextrose agar (PDA) plates supplemented with 250 mg/L amoxicillin, which had been sterilized by filtration. To verify the effectiveness of the sterilization and prevent external contamination, negative control procedures were employed. In this step, surface-sterilized leaf pieces were pressed directly onto PDA plates without being cut or inoculated. Another set was transferred to a second PDA plate. If fungal or bacterial colonies appeared on these control plates, the corresponding experimental plates were excluded to ensure only true endophytic fungi were isolated. The inoculated plates were incubated at 25 °C for approximately 2 weeks, or until visible fungal growth developed. Pure cultures of *P. oxalicum* were obtained by repeatedly subculturing the emerging fungal colonies onto fresh PDA plates [9, 10].

### Molecular identification of the isolated endophytic fungi

Pure cultures of *P. oxalicum* were identified by 18S rRNA gene sequencing [11]. Total fungal DNA was extracted and purified using the E.Z.N.A.^®^ Fungal DNA Mini Kit (D3390-01, Omega BIO-TEK, USA) according to the manufacturer’s protocol. The sequence of the utilised primer was 5′-CCTGGTTGATCCTGCCAGTA-3′ in the forward direction and 5′-GCTTGATCCTTCTGCAGGTT-3′ in the reverse direction. The sequences of the amplified products were determined at Macrogen Co., Korea. Then, the resulting sequences were put in the Gene Bank (https://blast.ncbi.nlm.nih.gov/Blast.cgi). We used BLAST tool to detect the sequence homology with the closest fungal isolates. Using MEGA 7.0 program, a phylogenetic tree was constructed.

### Preparation of *P. oxalicum* extract

A small amount of the purified *P. oxalicum* culture was inoculated into ten pre-sterilized one liter conical flasks, each containing 100 g of wheat and 110 mL of sterile water. The flasks were incubated at room temperature, in the dark, for 4 weeks to allow fungal growth. Following incubation, secondary metabolites were extracted using ethyl acetate (3 × 1 L). The extraction process involved sonication at 50 °C for 15 min, after which the extracts were filtered and pooled. The combined ethyl acetate extracts were then concentrated under reduced pressure using a rotary evaporator at 40 °C until a thick, semi-solid residue was obtained. The concentrated extract was air-dried to remove any remaining solvent before being stored for further analysis.

### Metabolomics analysis of *P. oxalicum* extract by LC–MS/MS

Metabolomics study of the extract of *P. oxalicum* was carried out according to the previously published methods [12, 13]. ExionLC™ AC UHPLC system (AB SCIEX, Concord, Canada) with Acquity XSelect HSS T3 analytical column 2.1 × 150 mm, 2.5 μm (Waters Co, Milford, US) sustained at 40 °C, combined with Triple TOF 5600+ mass spectrometer (AB SCIEX, Concord, Canada) was used for the Ultra-high-performance liquid chromatography high-resolution mass spectrometry (UHPLC-MS/MS) analysis. In the chromatographic separation, 10 µL of each sample was injected for 35 min employing gradient elution at a steady flow rate of 0.3 mL/min. Solutions of the mobile phase are as follows: solution A, 5 mM ammonium formate in 1% methanol at pH 3.0 for positive mode elution, solution B; 5 mM ammonium formate in 1% methanol at pH 8.0 for negative mode elution and solution C, acetonitrile 100%. Gradient elution was designed as follows: 0% C for 1.0 min, 0–90% C in 20 min, 90% for 4.0 min, 90–0% C in 1.0 min, and finally 3.0 min of re-equilibration with 0% C. The positive-ion (ESI+) and negative-ion (ESI) modes of mass spectrometric analysis were performed using a DuoSprayTM ion source [14].

Acquisition of Information Dependent Acquisition (IDA) with dynamic background subtraction was performed to detect the metabolites within the samples by setting TOF -MS scan from 50 to 1000 Da accumulated in 30 ms, followed by MS/MS on the 15 most intense precursor ions from 50 to 1000 Da using a fixed 50 Da transition window. The accumulation time for each of the MS/MS acquisitions was 50 ms, and collision energies were 35 V and − 35 V for the positive and negative modes, respectively. The overall cycle time was 0.6502 s. Analyst TF (v 1.7.1) was used to acquire MS and MS/MS spectra [15].

The MS-DIAL 4.9.2 platform was used to identify the metabolites in this study [16]. Global Natural Product Social Molecular Networking (GNPS) was the applied search space. Manual curation was done with the help of PeakView 2.2 and MasterView 1.1 program packages from AB SCIEX to enhance confidence in the detected metabolites with identification of parents and fragment ions. Precursor ion XIC S/*N* >10, sample: blank >5 were used as search criteria, and a mass tolerance of precursor of 10 ppm was applied.

### Preparation of oily core PEGylated PLGA NCs loaded with *P. oxalicum* extract

Oily core nanocapsules loaded with the ethyl acetate *P. oxalicum* extract were prepared using a hybrid single o/w emulsion /nanoprecipitation method [17, 18] as shown in Figure S1. The oily core is composed of PEG-PLGA (at different concentrations of 0.75, 1.5, and 3% w/v), Span 80 (2% w/v), and oleic acid (3.3% w/v) dissolved in a mixture of acetone, DMF, and DCM with a ratio of 1:1:1. Then the *P. oxalicum* extract (6.7% w/v) was added gradually during stirring till complete clarity of the internal oily phase. The oily drops were progressively added to the aqueous phase (Tween 80, 3% w/v, and PG, 10% w/v) using an ultrasonic probe sonication, which was adjusted at 35% of its highest power [18]. The oily: aqueous phase volume ratio was kept at 0.4:1. The organic phase was evaporated upon stirring for three hours. Then, the nanodispersion was washed with distilled water triplicate using a Hettich microliter centrifuge (MIKRO 220, Kirchlengern, Germany) at 10,000 RPM and − 4 °C for 10 min per cycle. The pellets of NCs loaded with the *P. oxalicum* extract were collected for further characterization.

### Characterization of PEGylated PLGA NCs loaded with the *P. oxalicum* extract

The effect of PEG-PLGA concentration, at three levels (Table 4), has been investigated in terms of surface charge, size distribution, and particle size of the prepared NCs. Also, morphological analysis has been examined.

### Detection of particle size, surface charge, and polydispersity index (PDI)

The Malvern Zetasizer Nano-zs90 (Malvern Instruments Ltd., Worcestershire, UK) has been utilized to determine the diameter (nm), zeta potential (mV), and the size distribution (PDI) of the prepared NCs. A sample of NCs (100 µL) has been optimally diluted with distilled water and assessed by the dynamic light scattering technique (DLS) for particle size and PDI analysis. Also, monitoring the electrophoretic mobility has been examined to measure the zeta potential [19]. The temperature has been kept at 25 °C during analysis, and all results have been displayed as mean ± SD for at least three measurements.

### Morphological examination

A transmission electron microscope (TEM, JEM-2100 Electron Microscope, JEOL Ltd., Akishima, Japan) was employed to reveal the morphology of NCs loaded with the *P. oxalicum* extract. All formulae were put on a carbon grid and then covered with carbon film till complete drying. After imaging, the diameter of NCs was recorded using the ImageJ (Bethesda, MD).

### In vitro antifungal action

Agar well diffusion technique was used to explore the antifungal action of the fungal extract and the PEGylated PLGA NCs loaded with the *P. oxalicum* extract on 10 *C. albicans* clinical isolates (obtained from the microbiology and immunology department culture collection, Faculty of pharmacy, Tanta university). In brief, the fungal suspensions were spread on Sabouraud dextrose agar (SDA) surface plates. Wells were then cut in the SDA and filled with 100 µL of the fungal extract (10 mg/ mL) and the PEGylated PLGA NCs loaded with the *P. oxalicum* extract. Fluconazole and 10% dimethyl sulfoxide were added in each well as positive and negative controls, respectively. Lastly, the plates were incubated at 37 °C for 24 h and were checked for inhibition zone formation [20].

### In vivo antifungal activity

#### Animals

Thirty adult male Swiss albino mice were employed in our study. Their weights ranged from 22 to 25 gm. All the instructions of using laboratory animals were followed according to the research ethics committee of the faculty of pharmacy, Tanta University, Egypt, with approval number (TP/RE/8/25 p-001).

### Experiment

The animals were randomly divided into five groups (*n* = 6 per group):

- Group I (Negative control): Received normal saline (5 mL/kg, orally) once daily.
- Group II (Positive control): Infected intravenously (IV) with *Candida albicans* (0.1 mL in the tail vein) and received saline (5 mL/kg, orally) once daily.
- Group III (Blank-treated group): Infected with *C. albicans* (0.1 mL, IV) and treated orally with the blank formula (5 mL/kg) once daily.
- Group IV (Fungal extract-treated group): Infected with *C. albicans* (0.1 mL, IV) and treated orally with fungal extract (10 mg/mL; 5 mL/kg) once daily.
- Group V (PEGylated PLGA NCs-treated group): Infected with *C. albicans* (0.1 mL, IV) and treated orally with PEGylated PLGA nanocapsules loaded with *P. oxalicum* extract (5 mL/kg) once daily [21].

All treatments were continued for 14 consecutive days. At the end of the experiment, the mice were anesthetized and euthanized. The liver, kidney, and spleen were collected for histopathological and immunohistochemical analyses. The fungal burden in each organ was determined and expressed as colony-forming units (CFU) per gram of tissue.

### Histological and immunohistochemical studies

Liver, kidney, and spleen were removed, and representative longitudinal sections were fixed in 10% neutral-buffered formalin and routinely processed for paraffin embedding. Section (5 μm) were cut using a standard rotary microtome and then placed on glass slides for staining with hematoxylin and eosin (H&E) [22, 23]. For Immunohistochemical staining, paraffin-embedded sections of liver, kidney, and spleen (5 μm) were dewaxed and rehydrated in a descending alcohol series. The sections were treated with 0.01% hydrogen peroxide to block endogenous peroxidase activity. Antigen retrieval was performed using 0.01 mol/L citrate buffer (pH 6.0) in a microwave at 98 °C for 10 min. The sections were then blocked with 5% bovine serum albumin in a humidified chamber and incubated overnight at 4 °C with an anti-TNF-α antibody (ab220210) and COX-2 antibody [NB100-689]. The immunostaining was visualized using diaminobenzidine for five minutes at room temperature. The sections were counterstained with hematoxylin for 10 min. Finally, the slides were cover-slipped and examined with an Olympus BX 50 automated light microscope.

### In silico studies

The protein data bank provided the crystal structure of the *C. albicans*’ aspartic proteinase 5 (SAP5) protein (PDB: 2QZX [24]), whereas the structures of SAP4 and SAP6 were Alphafold-models with the identity codes AF-Q5A8N2-F1 and AF-P43095-F1, respectively. The primary components of the extract, such as linoleic acid (LA), sinapinic acid (SA), alternariol monomethyl ether (AME), ellagic acid (EA), and kaurenic acid (KA), were subjected to a docking investigation using AutoDock Vina [25] in conjunction with the cocrystal inhibitor pepstatin. Marvin Sketch V22.2 [26] was used to create ligand structures, and the conformer with the highest energy preference was exported as a (*.pdb) file. Water molecules were eliminated, and AutoDock techniques were used to introduce hydrogen and apply gastier charges. For each receptor, Table 1 displays the centres and dimensions of the grid boxes that constitute the active site. The 2D schematic presentation and 3D visualization were produced using BIOVIA Discovery Studio Visualizer [27].

**Table 1:** Target proteins in *C. albicans*, their PDB code and grid box information

Protein	SAP4	SAP5	SAP6
Ref code	AF-Q5A8N2-F1	2QZX [24]	AF-P43095-F1
Grid coordinates (x, y, z)	− 5.8, − 8.3, − 5.7	8.2, 34.6, 26.9	− 9.4, 2.4, − 12.7
Grid Size (x, y, z)	17.7, 16.2, 15.8	28.9, 20.3, 21.2	28.3, 19.1, 21.8

### Statistics

Results were revealed as mean ± SD. The variation between the experimental groups was assessed by ANOVA followed by the post hoc test (Tukey). The alteration was regarded to be significant at *p* < 0.05 employing Prism version 8 (GraphPad Software, USA).

## Results

### Identification of the isolated endophytic fungi

Among the ten endophytic fungi isolated from *Acalypha hispida*, this fungal isolate was selected based on its distinctive morphological features and strong antifungal activity. The colony appeared fast-growing, velvety to powdery with bluish-green pigmentation on the surface (Figure S2). Microscopic examination revealed brush-like conidiophores with metulae and phialides bearing globose conidia in chains characteristic of the genus *Penicillium*. Molecular analysis was achieved using 18S rRNA gene sequencing, as illustrated in Figure S3. The DNA sequence was submitted to the database under the accession number ON100823, as shown in Table 2.

**Table 2:** Identification data of *P. oxalicum* by 18S rRNA sequencing

Identification	Accession number	Highly similarity Isolates	Highly similarity isolates accession number	Identity %
*Penicillium oxalicum*	ON100823	*Penicillium oxalicum* isolate QN0627.1 internal transcribed spacer 1, partial sequence; 5.8 S ribosomal RNA gene and internal transcribed spacer 2, complete sequence; and large subunit ribosomal RNA gene, partial sequence	MN103553.1	99.45

### Metabolomics studies

**Fig. 1:**
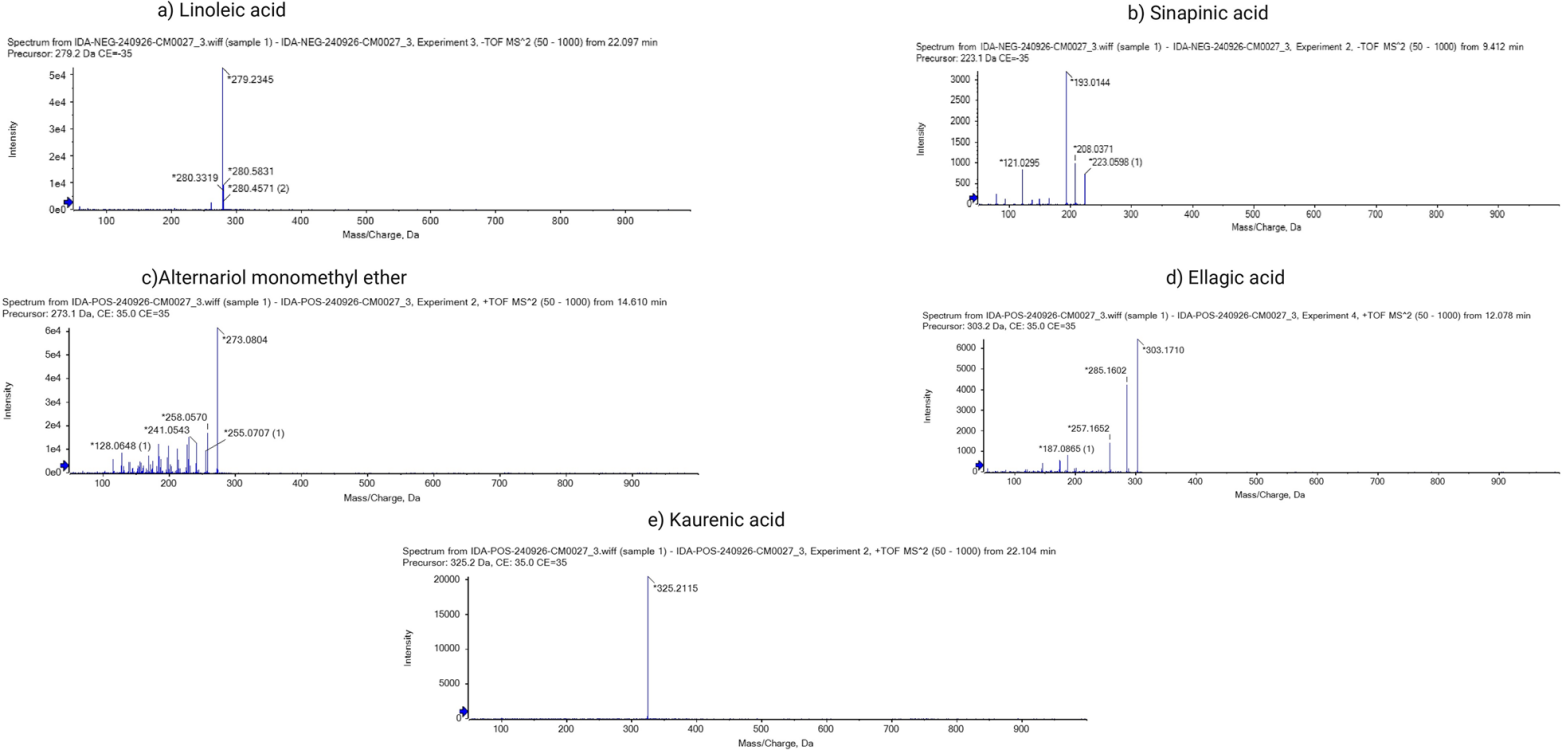
Mass spectra showing the fragmentation pattern for some of the selected metabolites. **a** Linoleic acid, **b** Sinapinic acid, **c** Alternariol monomethyl ether, **d** Ellagic acid, and **e** Kaurenic acid

In total, 32 compounds were tentatively determined in the extract of *P. oxalicum* by manual curation using UHPLC-MS/MS in positive and negative modes. Long-chain fatty acids, coumarins, diterpenoids *O*-glycosyl compounds, fatty acyl glycosides of mono and disaccharides, furofurans, eudesmane, isoeudesmane, and cycloeudesmane sesquiterpenoids constituted the major metabolites, as listed in Table 3. Figure S4 shows the total ion chromatography (TIC) of the *P. oxalicum* extract in positive and negative modes, while Fig. 1 depicts the mass spectra indicating the fragmentation pattern of the most abundant metabolites.

**Table 3:** A list of potentially identified metabolites in *Penicillium oxalicum* extract analyzed by UHPLC/MS

No.	Rt (min)	Error ppm	m/z	Adduct ion	Formula	Identification	Ontology	Structure
1	1.18	0.3	104.1067	[M+H]^+^	C_4_H_9_NO_2_	2-Amino-2-methylpropanoate	Alpha amino acids	
2	1.20	0.5	381.0798	[M+Na]^+^	C_16_H_22_O_9_	β-d-Glucopyranoside, 2-(1,3-benzodioxol-5-yl)-3-hydroxypropyl	*O*-glycosyl compounds	
3	1.63	0.4	111.0087	[M−H]−	C_4_H_4_N_2_O_2_	Uracil	Pyrimidones	
4	4.98	1	145.0503	[M−H]−	C_6_H_10_O_4_	3-Methyglutaric acid	Methyl-branched fatty acids	
5	5.58	− 0.4	312.1411	[M+H]^+^	C_18_H_17_NO_4_	8-Oxoerythraline	Erythrinanes	
6	5.96	− 0.8	141.0544	[M+H]^+^	C_7_H_8_O_3_	4-Hydroxy-3,6-dimethylpyran-2-one	Pyranones and derivatives	
7	6.70	0.2	162.0550	[M+H]^+^	C_6_H_11_NO_4_	*N*-methyl-l-glutamate	Glutamic acid and derivatives	
8	7.22	3.6	495.2772	[M+Na]^+^	C_22_H_32_O_11_	β-D-Glucopyranoside, 4-hydroxy-3-(3-methyl-2-buten-1-yl)phenyl 6-*O*-[(2R,3R,4R)-tetrahydro-3,4-dihydroxy-4-(hydroxymethyl)-2-furanyl]-	Phenolic glycoside	
9	7.59	0.4	275.0864	[M+Na]^+^	C_15_H_24_O_3_	2-Naphthaleneacetic acid, decahydro-8-hydroxy-4a,8-dimethyl-alpha-methylene (ilicic acid)	Eudesmane, isoeudesmane or cycloeudesmane sesquiterpenoids	
10	8.55	− 0.1	311.1029	[M+H]^+^	C_17_H_14_N_2_O_4_	Spiro[3 H-1,4-benzodiazepine-3,2ʹ-oxirane]-2,5(1 H,4 H)-dione, 3ʹ-(3-hydroxyphenyl)-4-methyl-, (3 S,3ʹR) (Cyclopenol)	1,4-benzodiazepines	
11	9.24	− 0.2	348.1893	[M+Na]^+^	C_20_H_23_NO_3_	Methyl *N*-(2,6-dimethylphenyl)-*N*-(phenylacetyl)alaninate (benalaxyl)	Alpha amino acid esters	
12	9.40	0.3	223.0611	[M−H]−	C_11_H_12_O_5_	Sinapinic acid	Hydroxycinnamic acids	
13	10.06	0.3	443.2033	[M+Na]^+^	C_22_H_28_O_8_	15-Acetoxyeremantholide B	Furofurans	
14	10.08	0.9	421.2211	[M+H]^+^	C_23_H_32_O_7_	6-[(2E,6E)-4,5-dihydroxy-4,6-dimethyl-7-(1,2,4-trimethyl-3,6-dioxabicyclo[3.1.0]hexan-4-yl)hepta-2,6-dien-2-yl]-4-methoxy-5-methylpyran-2-one	Polyketides	
15	10.46	1.1	254.0804	[M+H]^+^	C_15_H_11_NO_3_	3-Hydroxy-4-(3-hydroxyphenyl)-1 H-quinolin-2-one	Phenylquinolines	
16	11.67	− 0.2	259.0606	[M+H]^+^	C_14_H_10_O_5_	1,3,6-Trihydroxy-8-methylxanthen-9-one (Norlichexanthone)	Xanthones	
17	12.03	− 1.3	439.232	[M+Na]^+^	C_19_H_28_O_10_	2-Phenylethyl 2-*O*-[(2 S,3R,4R)-3,4-dihydroxy-4-(hydroxymethyl)tetrahydro-2-furanyl]-beta-d-glucopyranoside	O-glycosyl compounds	
18	12.06	− 0.1	303.1706	[M+H]^+^	C_14_H_6_O_8_	Ellagic acid	Hydrolyzable tannins	
19	12.49	− 0.9	351.1118	[M+Na]^+^	C_15_H_20_O_8_	3-(4-Hydroxyphenyl)-3-oxopropyl beta-d-glucopyranoside	Fatty acyl glycosides of mono- and disaccharides	
20	13.72	1	289.1771	[M+Na]^+^	C_15_H_22_O_4_	2,8-Dihydroxy-5,5,8-trimethyl-11-oxatetracyclo[7.3.1.0 ~ 1,9 ~ 0.0 ~ 3,7~]tridecan-10-one (Isolactarorufin)	Diterpenoids	
21	13.98	0.1	439.2081	[M+Na]^+^	C_19_H_28_O_10_	Benzyl 6-*O*-(6-deoxy-alpha-l-mannopyranosyl)-beta-d-glucopyranoside (hydrangeifolin I)	*O*-glycosyl compounds	
22	14.09	0.2	345.1707	[M+Na]^+^	C_20_H_34_O_3_	2-Butene-1,4-diol, 2-[2-[(1 S,8aR)-1,4,4a,5,6,7,8,8a-octahydro-2-(hydroxymethyl)-5,5,8a-trimethyl-1-naphthalenyl]ethyl]-, (2Z)-	Diterpenoids	
23	14.62	0.1	273.0757	[M+H]^+^	C_15_H_12_O_5_	Alternariol monomethyl ether	Coumarins and derivatives	
24	18.09	− 0.3	401.2324	[M+H]^+^	C_25_H_36_O_4_	Cyclocitrinol	Steroid acids	
25	21.40	− 0.9	399.3073	[M+Na]^+^	C_23_H_36_O_4_	2-Pentenoic acid, 3-[(acetyloxy)methyl]-5-[(1R,4aR,8aR)-decahydro-5,5,8a-trimethyl-2-methylene-1-naphthalenyl]-, methyl ester, (2Z)-	Terpenoid	
26	21.64	− 0.2	253.2173	[M−H]−	C_16_H_30_O_2_	Palmitoleic acid	Long-chain fatty acids	
27	22.04	0.1	353.2657	[M+Na]^+^	C_16_H_26_O_7_	(2,6,6-Trimethyl-4-oxo-2-cyclohexen-1-yl)methyl beta-d-glucopyranoside	*O*-glycosyl compounds	
28	22.09	0.5	325.2099	[M+Na]^+^	C_20_H_30_O_2_	Kaurenic acid	Kaurane diterpenoids	
29	22.10	1	279.2335	[M−H]−	C_18_H_32_O_2_	Linoleic acid	Lineolic acids and derivatives	
30	22.10	2.3	307.2239	[M+Na]^+^	C_14_H_20_O_6_	2-Phenylethyl beta-d-glucopyranoside	*O*-Glycosyl compounds	
31	23.28	− 0.3	281.2491	[M−H]−	C_18_H_34_O_2_	Petroselinic acid	Long-chain fatty acids	
32	25.58	0.9	413.2647	[M+Na]^+^	C_24_H_38_O_4_	2-Acetoxy-4-pentadecylbenzoic acid	Acylsalicylic acids	

### Effect of m-PEG-PLGA concentration on particle size of PEGylated PLGA NCs loaded with the *P. oxalicum* extract

**Fig. 2:**
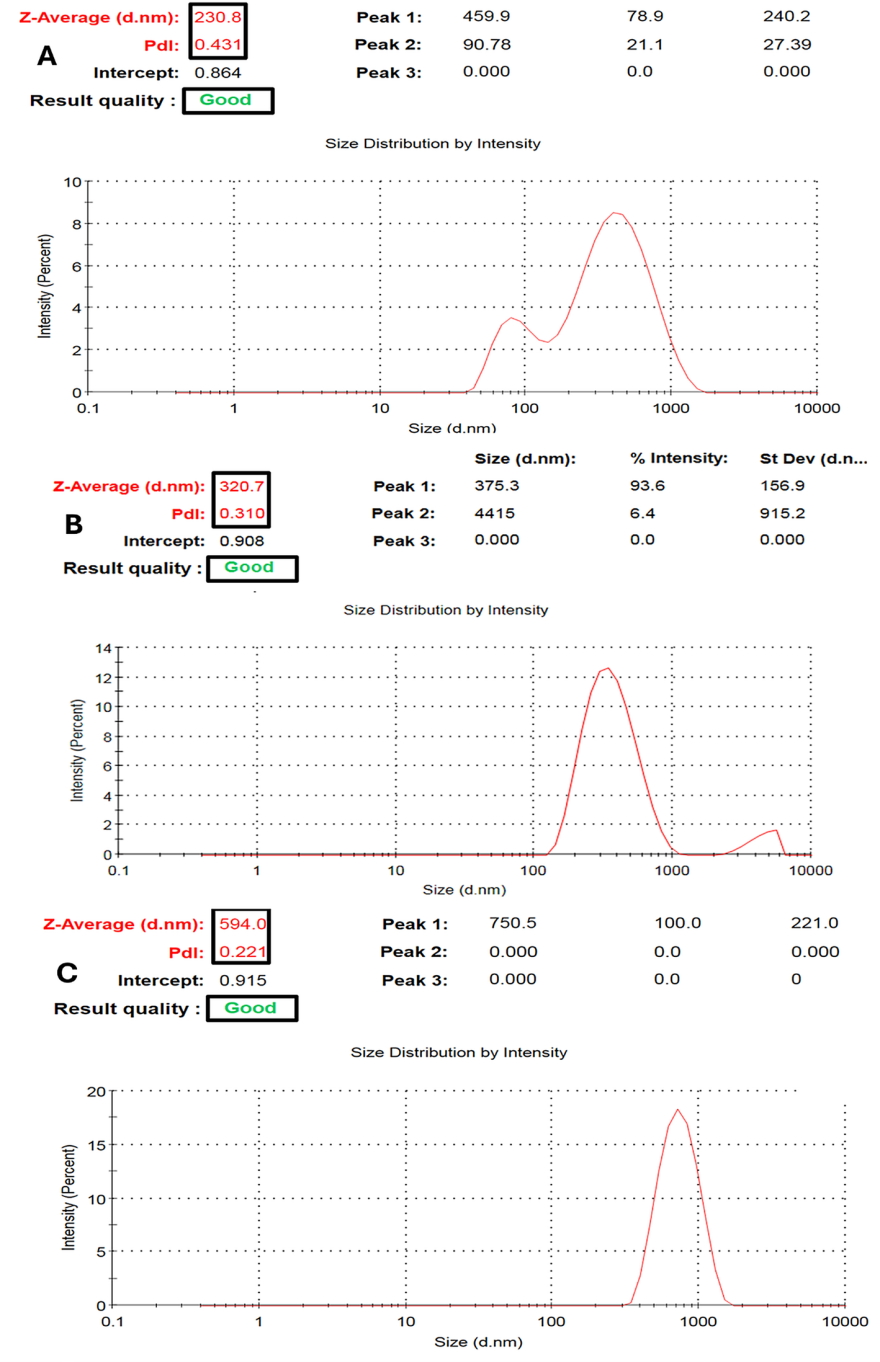
Charts displaying particle size and PDI measurement of PEGylated PLGA NCs loaded with the *P. oxalicum* extract. **A** F1, **B** F2, and **C** F3

**Fig. 3:**
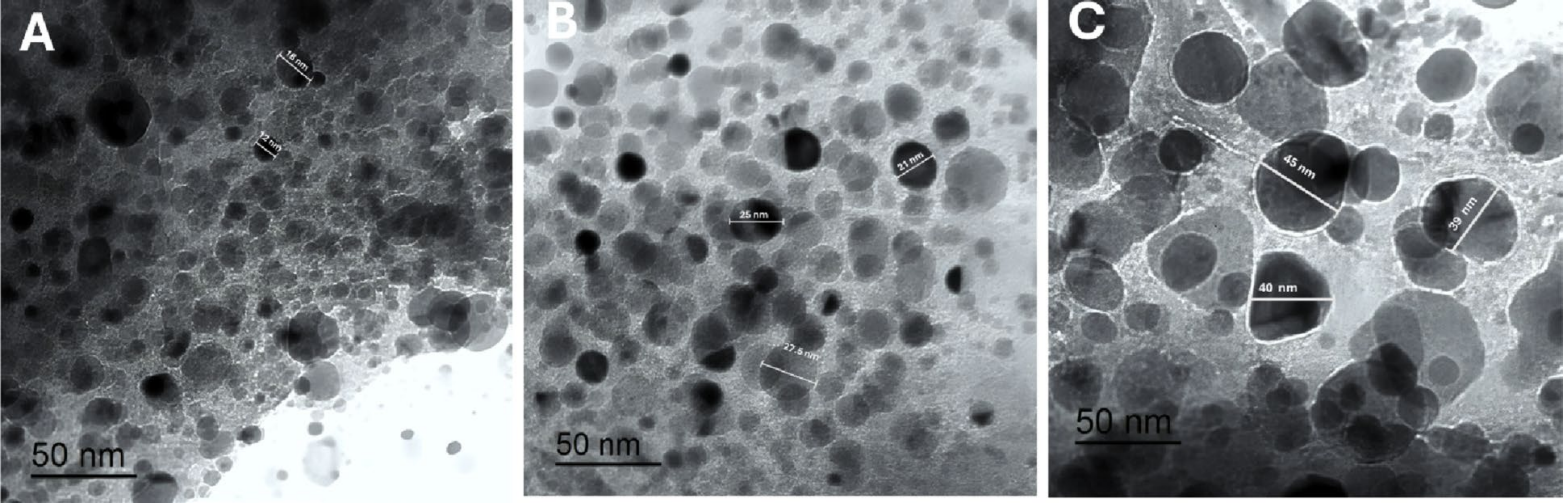
Transmission electron microscopy (TEM) images of PEGylated PLGA NCs loaded with the *P. oxalicum* extract. **A** F1, **B** F2, and **C** F3

Increasing m-PEG-PLGA concentration led to a noteworthy rise in the particle size of the prepared NCs (*p* < 0.05), as exposed in Table 4; Figs. 2 and 3.

**Table 4:** The physical properties of NCs loaded with the *P. oxalicum* extract

Formula Code	PEG-PLGA Conc. (%w/v)	Particle Size (nm)	Zeta Potential (mV)	PDI
F1	0.75	234.50 ± 4.09	− 16.53 ± 0.81	0.47 ± 0.038
F2	1.5	325.70 ± 4.38	− 24.57 ± 3.57	0.34 ± 0.044
F3	3	577.77 ± 14.77	− 29.47 ± 0.70	0.22 ± 0.042

Data represented as mean ± SD

**Fig. 4:**
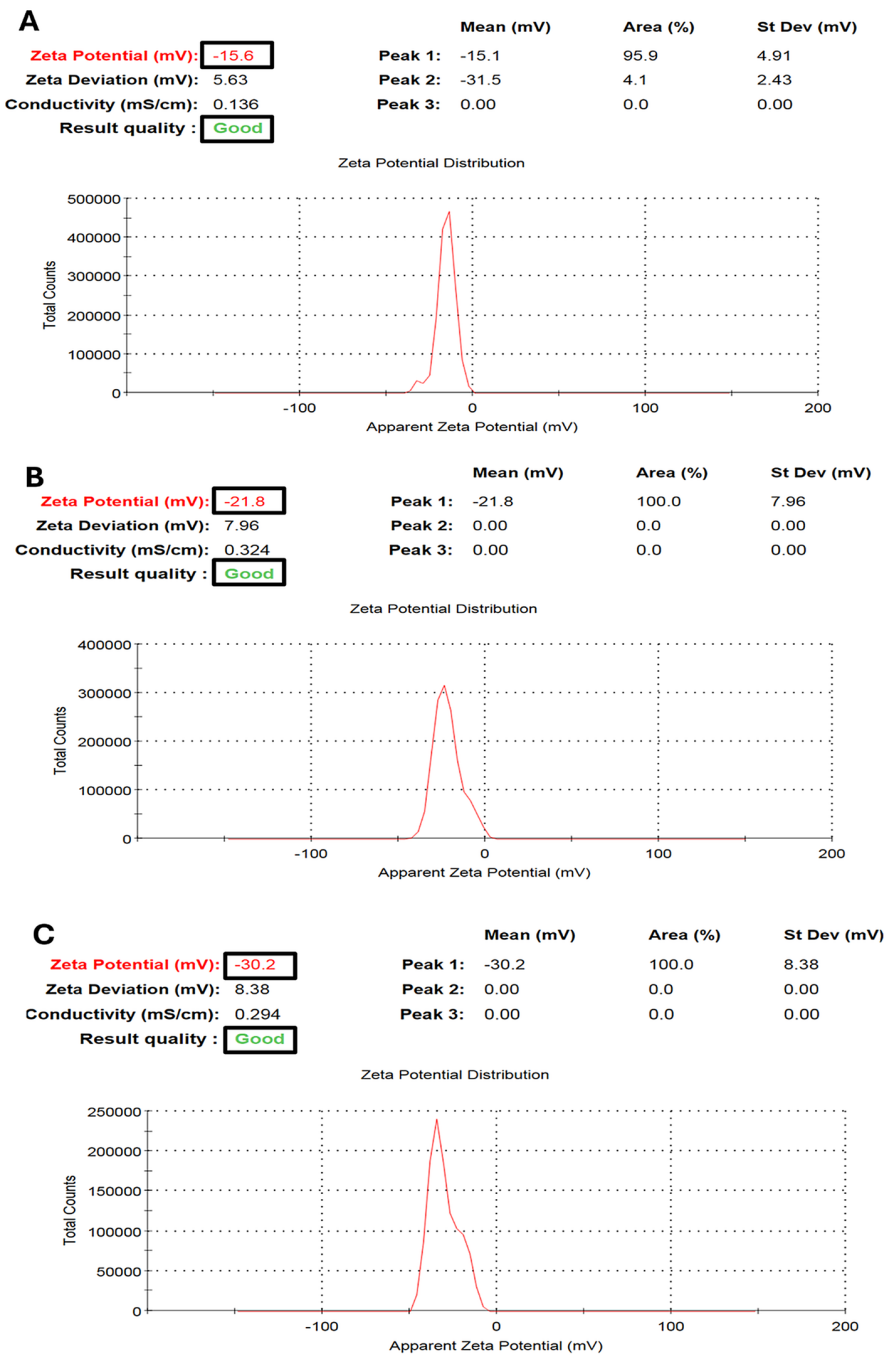
Representative charts showing zeta potential measurement of the PEGylated PLGA NCs loaded with the *P. oxalicum* extract. **A** F1, **B** F2, and **C** F3

At m-PEG-PLGA concentrations of 0.75, 1.5, and 3% w/v, the resulting NCs exhibited particle size equal to 234.50 ± 4.09, 325.70 ± 4.38, and 577.77 ± 14.77 nm for F1, F2, and F3, respectively. The morphological examination using TEM showed the spherical shape of the prepared NCs (Fig. 4) with an acceptable range of size deviation between the examined particles.

### Polydispersity index (PDI) of PEGylated PLGA NCs loaded with the *P. oxalicum* extract

PDI values lie in the accepted range (less than 0.5) for all the prepared PEGylated PLGA NCs loaded with the *P. oxalicum* extract. As shown in Fig. 3; Table 4, an inverse relation could be observed between the measurement of particle size and PDI. NCs with bigger particle size (F3, 577.77 ± 14.77 nm) displayed a low value of PDI (0.22 ± 0.042), whereas the smaller NCs (F1, 234.50 ± 4.09 nm) showed a significantly (*p* < 0.05) greater value of PDI (0.47 ± 0.038).

### Zeta potential of the PEGylated PLGA NCs loaded with the *P. oxalicum* extract

The negative values of the zeta potential increased at higher m-PEG-PLGA concentrations (Fig. 4; Table 4). At m-PEG-PLGA concentrations of 0.75, 1.5, and 3% w/v, the resulting NCs exhibited zeta potential values equal to − 16.53 ± 0.81, − 24.57 ± 3.57, and − 29.47 ± 0.70 mV for F1, F2, and F3 respectively.

### Antifungal action

Both the fungal extract and the prepared formula revealed antifungal activity on the tested isolates, and the inhibition zone diameters are illustrated in Figure S5. There was a significant increase (*p* < 0.05) in the diameters of the inhibition zones of the PEGylated PLGA NCs loaded with the *P. oxalicum* extract in relation to the fungal extract, which means a significant increase in the antifungal action (Figure S6). Regarding the in vivo antifungal action, there was a noteworthy lessening in the *C. albicans* count in the studied tissues in comparison with the positive control, blank, and the fungal extract groups (Figure S7).

**Fig. 5:**
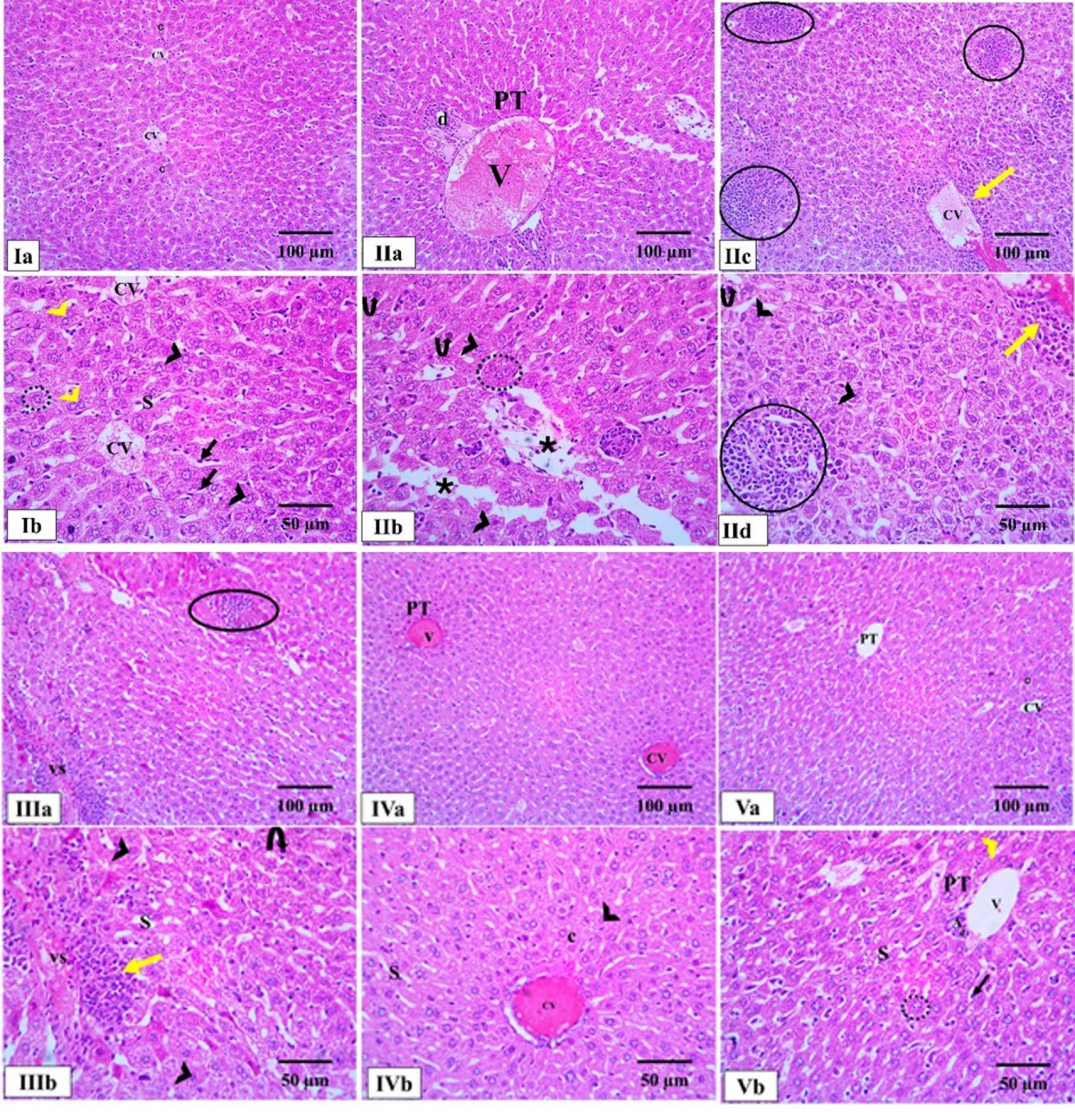
Photomicrographs of light microscopic analysis of liver architecture by H&E staining of all studied groups. **Ia**, **Ib** Normal control group showing a normal hepatic architecture in the form of hepatic lobules with hepatocytes radiating like cords (C) from the central veins (CV) to the periphery. The blood sinusoids (S) separated the plates of hepatocytes, which are lined with endothelial cells and Kupffer cells (black arrow). Hepatocytes (dashed circle) had granular eosinophilic cytoplasm with central vesicular nuclei and prominent nucleoli (arrowhead). Some hepatocytes are seen with two nuclei (yellow arrowhead). **IIa–IId** Positive control group showing a preserved hepatic architecture with inflammatory cell infiltrations (yellow arrow) around the dilated congested central veins and portal veins (V). Proliferation of bile ductules (d) is observed. Also, there are marked pericentral inflammatory cell infiltrations forming collections (circle). Most hepatocytes appeared with karyolytic nuclei (black arrowhead) and Kupffer cells engulfing hemosiderin granules (iron produced from RBCs destruction) were detected (curved arrow). Diffuse vacuolar degeneration of hepatocytes with multiple large vacuoles (V) and ballooned hepatocytes can be seen (dashed circle). **IIIa**, **IIIb** The blank group showing inflammatory cell infiltrations forming collections (circle). Most hepatocytes appear with karyolitic nuclei (black arrowhead), and Kupffer cells engulfing hemosiderin granules (iron produced from RBCs destruction) are detected (curved arrow). Dilated, congested venous sinusoids (VS) are noticed. **IVa**, **IVb** The *P. oxalicum* extract group showing a moderate improvement of the hepatic structure in the form of organized hepatic architecture with hepatic cords (c) radiating from the central vein (CV) and separated by the hepatic sinusoids (S). However, there are dilated, congested central vein (CV) and portal vein (V). **Va**, **Vb** The PEGylated PLGA NCs loaded with the *P. oxalicum* extract group showing a marked enhancement of hepatic structure, as the normal control group. An organized hepatic architecture is noticed with hepatic cords (c) radiating from the central vein (CV) and separated by the hepatic sinusoids (S) with pericentral zone and midzone hepatocytes. Polyhedral hepatocytes (dashed circle) appear with rounded vesicular nuclei and granular eosinophilic cytoplasm separated by sinusoids (S), which are lined with endothelial cells and Kupffer cells. At the periphery of the lobules, branches of the portal veins (V), hepatic arteries(A), and bile ducts (d) are seen (the portal triad) (H&E × 200, scale bar = 100 μm, H&E × 400, scale bar = 50 μm)

The livers in the normal control group showed a healthy and well-organized structure. The liver tissue was composed of hepatic lobules, with hepatocytes, arranged in cords that radiated outwards from a central vein. These cords were separated by sinusoids, which were lined with endothelial cells and specialized immune cells called Kupffer cells. The hepatocytes themselves had a granular, pink-staining cytoplasm and a prominent nucleus; some even have two nuclei. In contrast, the livers from the positive control and blank groups showed a significant damage. While the overall structure of the liver was somewhat preserved, there’s widespread inflammation, particularly around the swollen and congested central and portal veins. There was also an overgrowth of bile ductules, which could indicate an injury. Most of the hepatocytes showed signs of cell death, specifically karyolysis, where the nucleus has broken down. Additionally, the Kupffer cells were filled with hemosiderin granules, a byproduct of red blood cell destruction, indicating a breakdown of blood cells. The hepatocytes were also suffering from vacuolar degeneration, where they were swollen up with multiple large vacuoles, or empty spaces. The treatment groups showed varying degrees of recovery. The fungal extract group demonstrated moderate improvement, with the liver’s architecture becoming more organized. The cords of hepatocytes were once again radiating from the central vein, though some of the veins were still dilated and congested. The PEGylated PLGA NCs loaded with the *P. oxalicum* extract group showed the most significant recovery, with the liver structure appearing nearly identical to the normal control group. The well-organized hepatic cords and central veins were clear, and the hepatocytes looked healthy, with a normal shape, granular cytoplasm, and a prominent nucleus. The sinusoids were properly lined with endothelial and Kupffer cells, and the portal triad (the portal vein, hepatic artery, and bile duct) was also clearly visible and well-structured (Fig. 5).

**Fig. 6:**
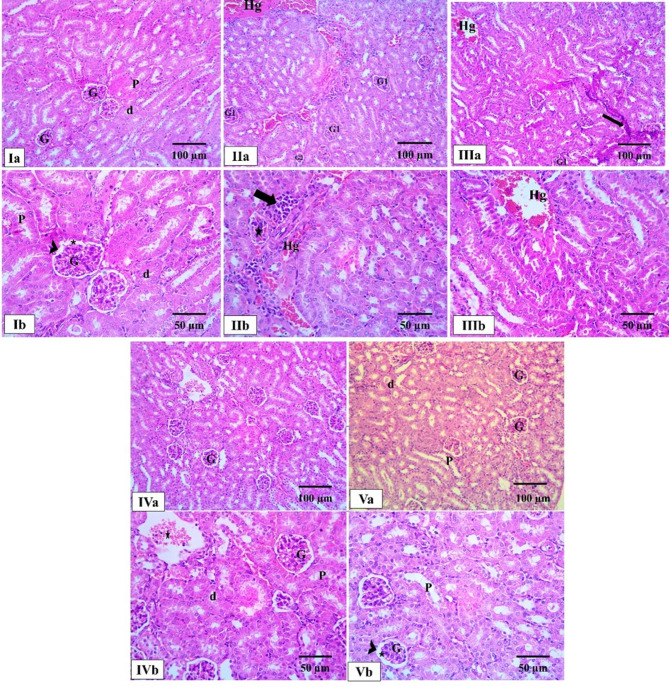
Photomicrographs of light microscopic analysis of kidney cortices sections by H&E staining of all studied groups. **Ia**, **Ib** the kidneys of the normal control group seeming completely normal. The renal corpuscles, are made up of Bowman’s capsule (arrowhead) that surrounds a little bundle of blood vessels, known as the glomerulus (G). The glomerulus is covered in special cells called podocytes. Also, the kidney’s proximal tubules (P) are very pink and have a fuzzy border. The distal tubules (d) have clearer, more open spaces inside and their cells don’t have that fuzzy border. **IIa–IId** In contrast, the kidneys of positive control group are disorganized and shows a significant damage. Some glomeruli are shrunken and wasted away (G2), while others are swollen with blood and congested (G1) with an increase in cellularity and an expressive reduction in Bowman’s spaces (star). The kidney tubules are severely damaged, with many cells showing signs of necrosis. These dead cells have tiny, dark, shrunken nuclei and are even sloughing off into the tubes. The spaces inside the tubules are also widened. There’s also bleeding (Hg) and an increase in inflammatory cells (black arrow) in the tissue between the tubules. **IIIa**, **IIIb** The blank group shows significant damage as positive control group. **IVa**, **IVb** The *P. oxalicum* extract group shows that most of the glomeruli (G) are healthy and returned to a normal state. However, some damage persisted in the kidney tubules. There were a few casts inside the tubules and empty spaces in the lining of the cells (star). **Va**,** Vb** The PEGylated PLGA NCs loaded with the *P. oxalicum* extract group shows marked enhancement giving normal renal cortical structure as normal control group (H & E × 200, scale bar = 100 μm & H & E × 400, scale bar = 50 μm)

The kidneys of the normal control group appeared healthy and normal. The main filtering units or the renal corpuscles were well-formed. Each one consisted of a Bowman’s capsule that enclosed a glomerulus, a tiny cluster of blood vessels. The glomerulus was properly covered by specialized cells called podocytes. The kidney’s tubules were also clearly distinguishable. Proximal tubules were characterized by their intense pink color and a distinct fuzzy border, while distal tubules had a clearer, more open interior and lack this border. In stark contrast, the kidneys from the positive control and blank groups showed an extensive damage and disorganization. The glomeruli were severely affected, with some being shrunken and atrophied while others were swollen with blood and congested. There was an increase in cellularity and a noticeable reduction in the space within Bowman’s capsule. The kidney tubules were in very poor condition, showing widespread cell death, or necrosis. These dead cells had shrunken, dark nuclei and were detaching into the tubes’ interior, which was also widened. Additionally, there was bleeding and an increased presence of inflammatory cells in the tissue surrounding the tubules. Both treatment groups showed signs of recovery. In the *P. oxalicum* extract group, most of the glomeruli appeared healthy and had returned to a normal state. However, some damage lingered in the kidney tubules, evidenced by the presence of casts (small clumps of material) and empty spaces within the lining of the cells. The PEGylated PLGA NCs loaded with the *P. oxalicum* extract group demonstrated the most significant improvement, with the kidney’s cortical structure appearing completely normal and well-organized, almost identical to that of the healthy control group. This indicates that the formulated drug provided a marked enhancement and recovery of the kidney tissue (Fig. 6).

**Fig. 7:**
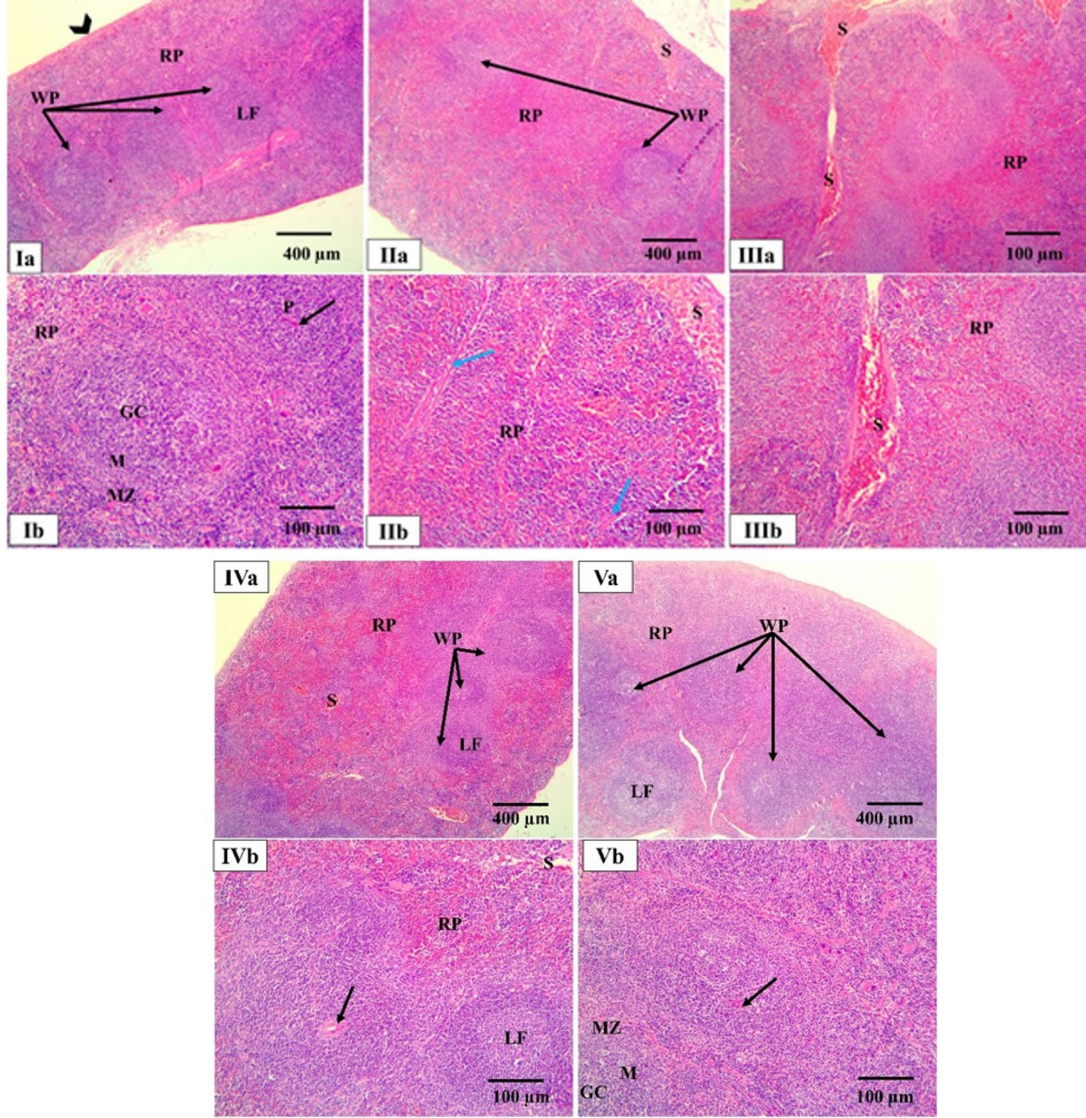
Photomicrographs of light microscopic analysis of splenic sections by H&E staining of all studied groups. **Ia**, **Ib** Normal control group showing a normal histological organization of white pulp (WP) and red pulp (RP) surrounded by capsule (arrowhead). The WP presents the central arteriole (black arrow) and lymphoid follicles (LF) with a germinal centers (GC) and mantle region (M) surrounded by a loosely distributed marginal zone (MZ). The RP presents lymphocytes, trabeculae, and sinusoids. **IIa–IId** The positive control group showing a disorganized architecture and shrunken WP and broadened RP with total loss of the delineation of the splenic nodules. Congested dilated splenic sinuses (S) are seen in the RP. Many cells in the WP appear vacuolated. Thick fibrous trabeculae are observed (blue arrow). **IIIa**, **IIIb** The blank group showing a disorganized splenic architecture with similar features to the positive control group. **IVa**, **IVb** The *P. oxalicum* extract group showing a nearly normal appearance of the splenic architecture. However, dilated congested splenic sinus is also seen. **Va**, **Vb** The PEGylated PLGA NCs loaded with the *P. oxalicum* extract group showing a nearly normal appearance of the splenic architecture of WP and RP as the normal control group (H&E × 100, scale bar = 200 μm, H&E × 200, scale bar = 100 μm)

The spleen from the normal control group exhibited a healthy and well-organized structure. The spleen was encased in a capsule and is clearly divided into two distinct areas: the white pulp (WP) and the red pulp (RP). The WP, which is the site of immune activity, contained lymphoid follicles with a central arteriole, as well as distinct germinal centers and mantle regions. The RP, which filters blood, was characterized by lymphocytes, trabeculae, and sinusoids. In contrast, the positive control and the blank groups showed a significant disorganization and damage. The architecture was severely disrupted, with the WP appeared shrunken and the RP was broader. There was a complete loss of the normal structure of the splenic nodules. The RP showed dilated and congested splenic sinuses. Additionally, many cells in the WP appeared with empty spaces (vacuoles), and there was a thickening in the fibrous trabeculae. Both treatment groups showed signs of recovery. The *P. oxalicum* extract group had a nearly normal spleen appearance, but there was still some lingering evidence of damage, specifically the presence of dilated and congested splenic sinuses. The PEGylated PLGA NCs loaded with the *P. oxalicum* extract group showed the most significant improvement, with the spleen’s architecture appearing almost completely normal, very similar to the normal control group. This indicates that the formulated drug was highly effective in restoring the normal structure of both the WP and RP (Fig. 7).

**Fig. 8:**
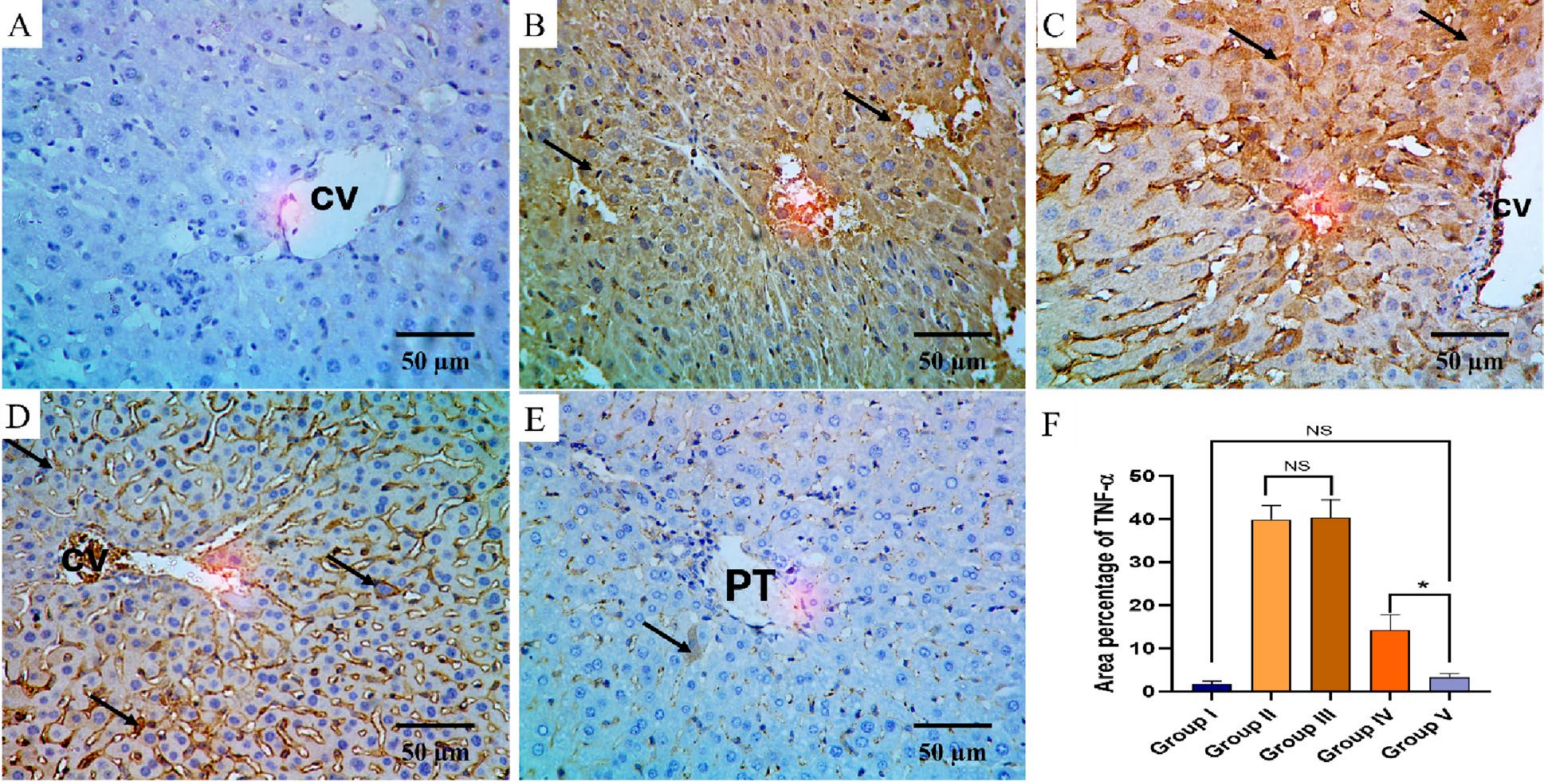
TNF-α immuno-histochemical staining analysis of liver section in various studied groups: **A** The normal control group express a negative TNF-α reaction. **B**, **C** The positive control and blank groups express a strong positive TNF-α reaction in the form of brown stain within the cytoplasm of hepatocytes (arrows) with a significant difference with both the normal control group and the PEGylated PLGA NCs loaded with the *P. oxalicum* extract group. **D** The *P. oxalicum* extract group expresses a moderate TNF-α reaction. **E** The PEGylated PLGA NCs loaded with the *P. oxalicum* extract group express few TNF-α positive cells (arrow) and negative reaction in the most of cells with non-significant difference with the normal control group. **F** Area percentage of TNF-α immune reaction in all groups. The single asterisk denotes a significant (*p* < 0.05) difference, and the abbreviation (NS) denotes a non-significant (*p* > 0.05) difference (TNF-α × 400, scale bar = 50 μm)

**Fig. 9:**
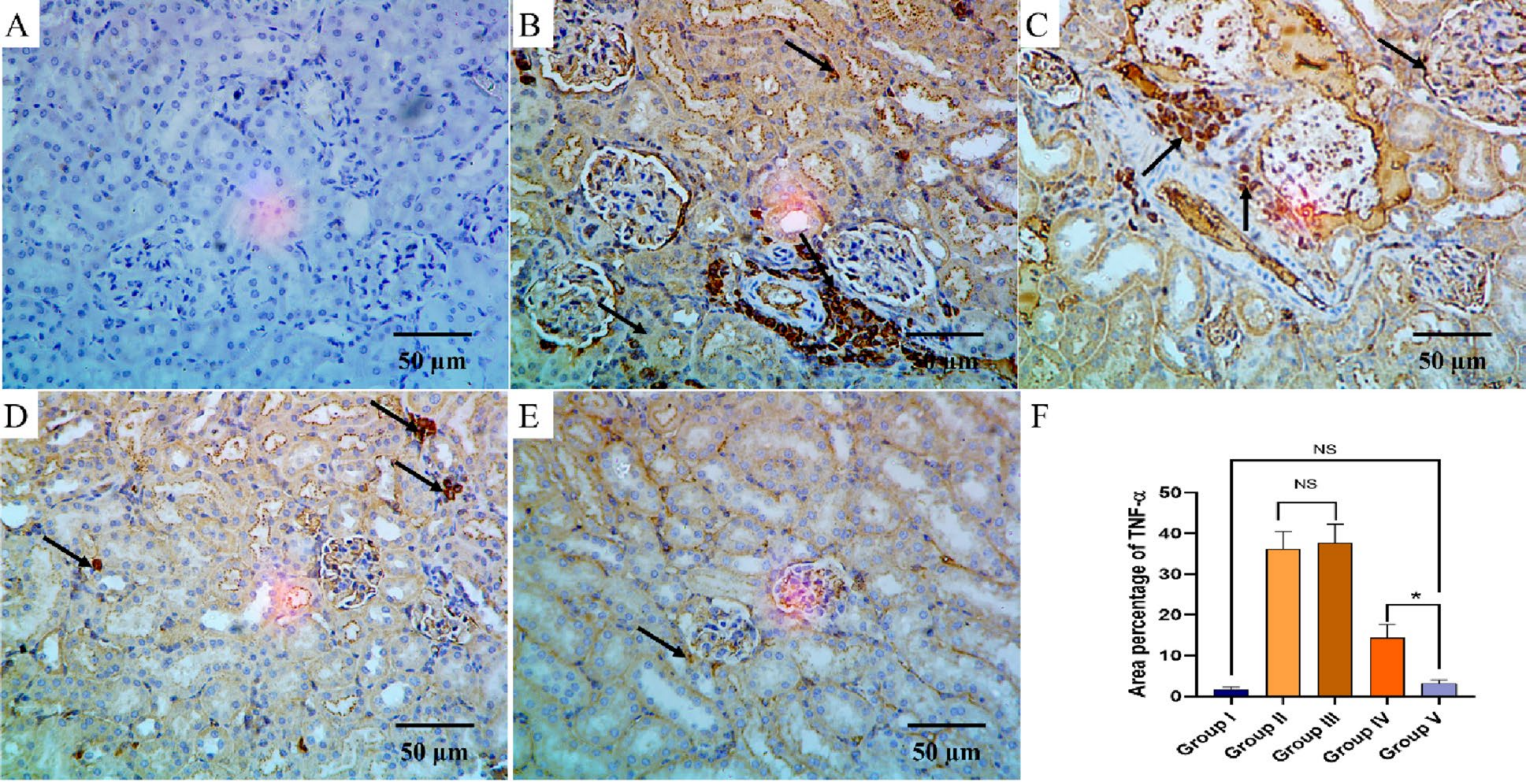
TNF-α immuno-histochemical staining analysis of kidney section in various studied groups: **A** The normal control group express a negative TNF-α reaction. **B**, **C** The positive control and blank groups express a strong positive TNF-α reaction in the form of brown stain within the cytoplasm (arrows) with significant difference with both control group and the PEGylated PLGA NCs loaded with the *P. oxalicum* extract group. **D** The *P. oxalicum* extract group express a moderate TNF-α reaction. **E** The PEGylated PLGA NCs loaded with the *P. oxalicum* extract group express few TNF-α positive cells (arrow) and a negative reaction in most of cells with non-significant difference with the normal control group. **F** Area percentage of TNF-α immune reaction in all groups. The single asterisk denotes a significant (*p* < 0.05) difference, and the abbreviation (NS) denotes a non-significant (*p* > 0.05) difference (TNF-α × 400, scale bar = 50 μm)

**Fig. 10:**
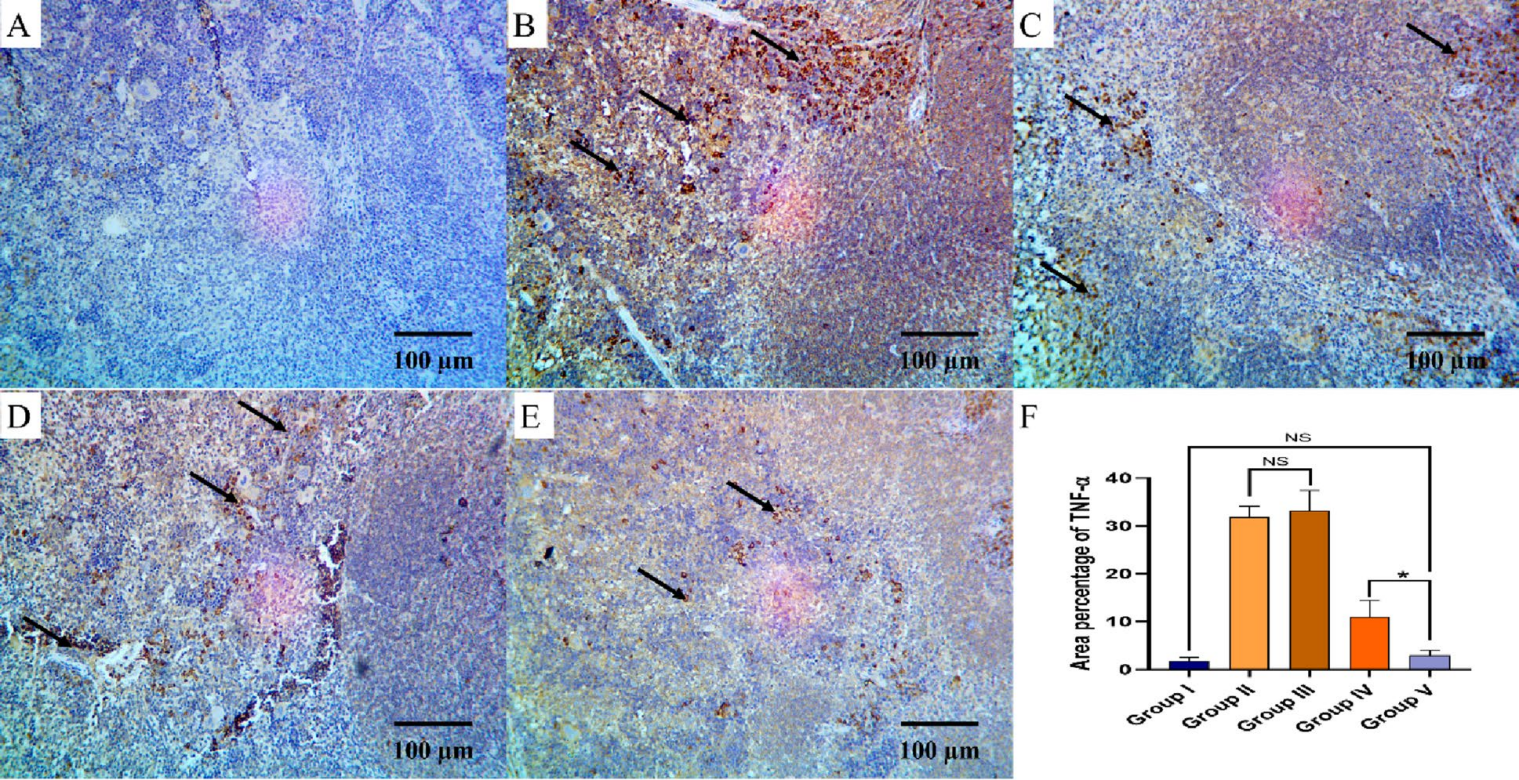
TNF-α immuno-histochemical staining analysis of splenic section in various studied groups: **A** The normal control group express a negative TNF-α reaction. **B**, **C** The positive control and blank groups express a strong positive TNF-α reaction in the form of brown stain within the cytoplasm (arrows) with a significant difference with both control group and the PEGylated PLGA NCs loaded with the *P. oxalicum* extract group. **D** The *P. oxalicum* extract group express a moderate TNF-α reaction. **E** The PEGylated PLGA NCs loaded with the *P. oxalicum* extract group express few TNF-α positive cells (arrow) and a negative reaction in most of cells with non-significant difference with normal control group. **F** Area percentage of TNF-α immune reaction in all groups. The single asterisk denotes a significant (*p* < 0.05) difference, and the abbreviation (NS) denotes a non-significant (*p* > 0.05) difference (TNF-α × 400, scale bar = 50 μm)

**Fig. 11:**
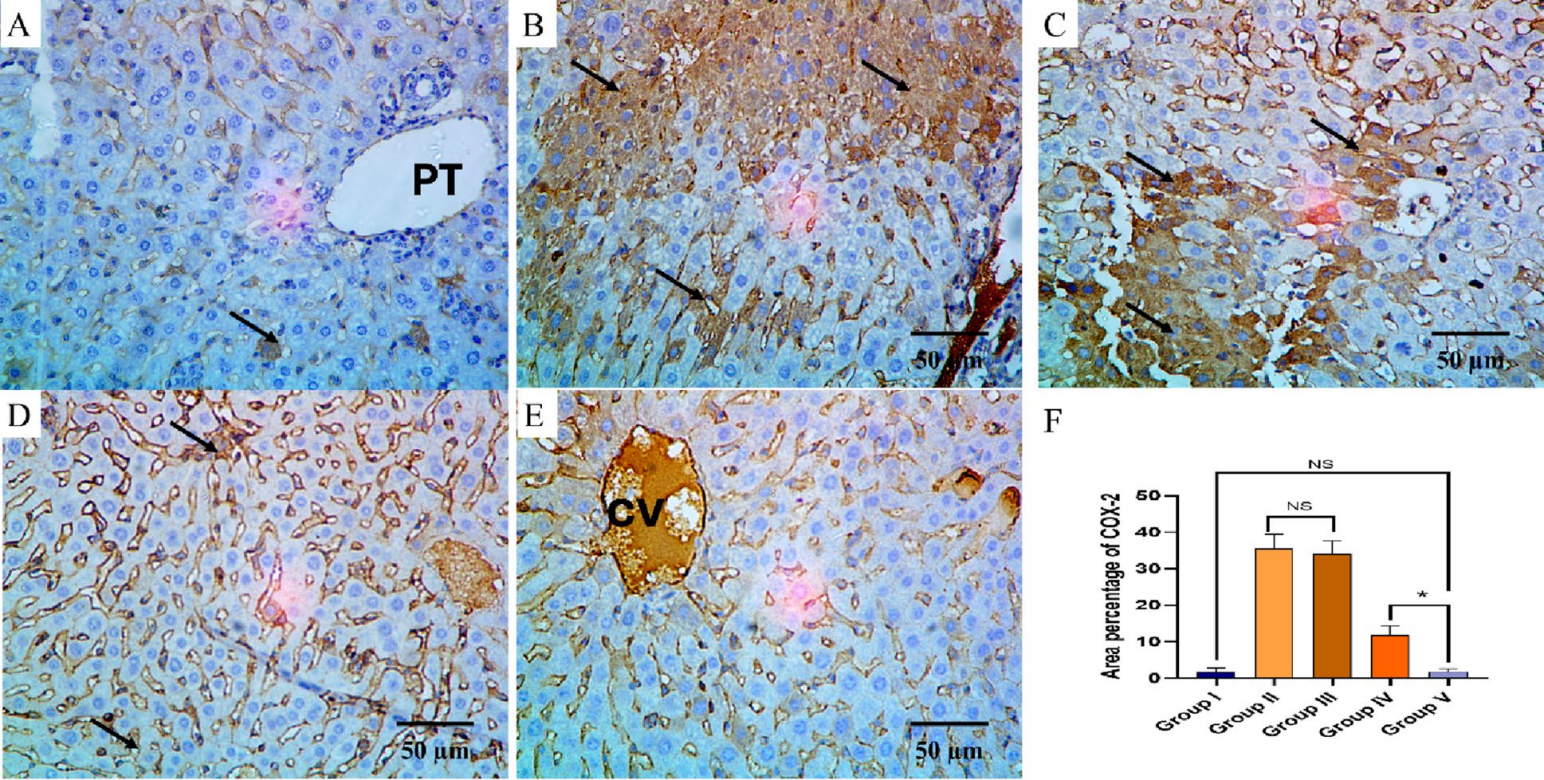
COX-2 immuno-histochemical staining analysis of liver section in various studied groups: **A** The normal control group expresses a negative COX-2 reaction. **B**, **C** The positive control and blank groups express a strong positive COX-2 reaction in the form of brown stain within the cytoplasm of hepatocytes (arrows) with significant difference with both control group and the PEGylated PLGA NCs loaded with the *P. oxalicum* extract group. **D** The *P. oxalicum* extract group expresses a moderate COX-2 reaction. **E** The PEGylated PLGA NCs loaded with the *P. oxalicum* extract group express few COX-2 positive cells (arrow) and a negative reaction in most of cells with non-significant difference with the normal control group. **F** Area percentage of COX-2 immune reaction in all groups. The single asterisk denotes a significant (*p* < 0.05) difference, and the abbreviation (NS) denotes a non-significant (*p* > 0.05) difference (COX-2 × 400, scale bar = 50 μm)

**Fig. 12:**
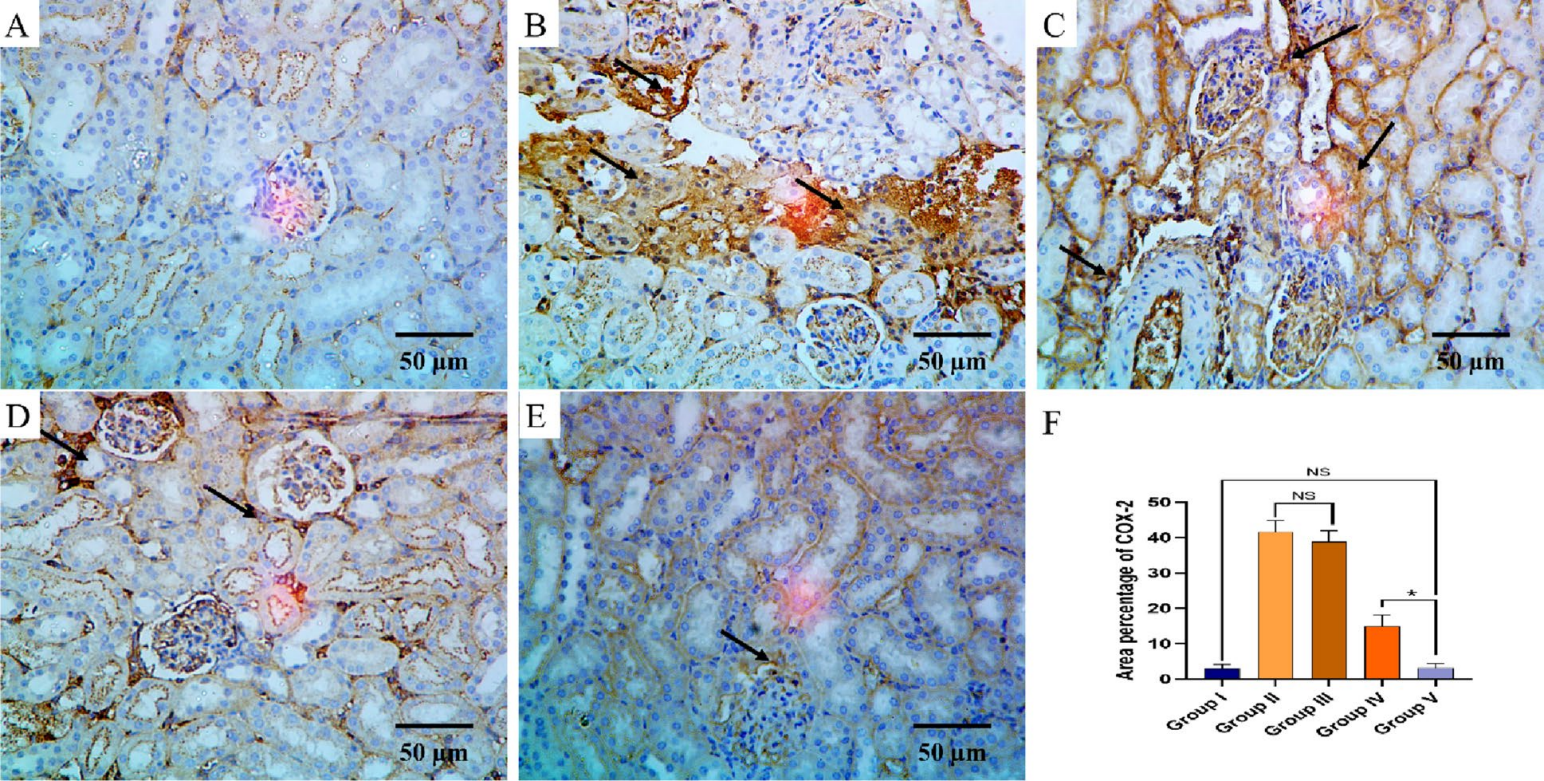
COX-2 immuno-histochemical staining analysis of kidney section in various studied groups: **A** The normal control group express a negative COX-2 reaction. **B**, **C** The positive control and blank groups express a strong positive COX-2 reaction in the form of brown stain within the cytoplasm (arrows) with a significant difference with both control group and the PEGylated PLGA NCs loaded with the *P. oxalicum* extract group. **D** The *P. oxalicum* extract group expresses a moderate COX-2 reaction. **E** The PEGylated PLGA NCs loaded with the *P. oxalicum* extract group express few COX-2 positive cells (arrow) and a negative reaction in most of cells with a non-significant difference with normal control group. **F** Area percentage of COX-2 immune reaction in all groups. The single asterisk denotes a significant (*p* < 0.05) difference, and the abbreviation (NS) denotes a non-significant (*p* > 0.05) difference (COX-2 × 400, scale bar = 50 μm)

**Fig. 13:**
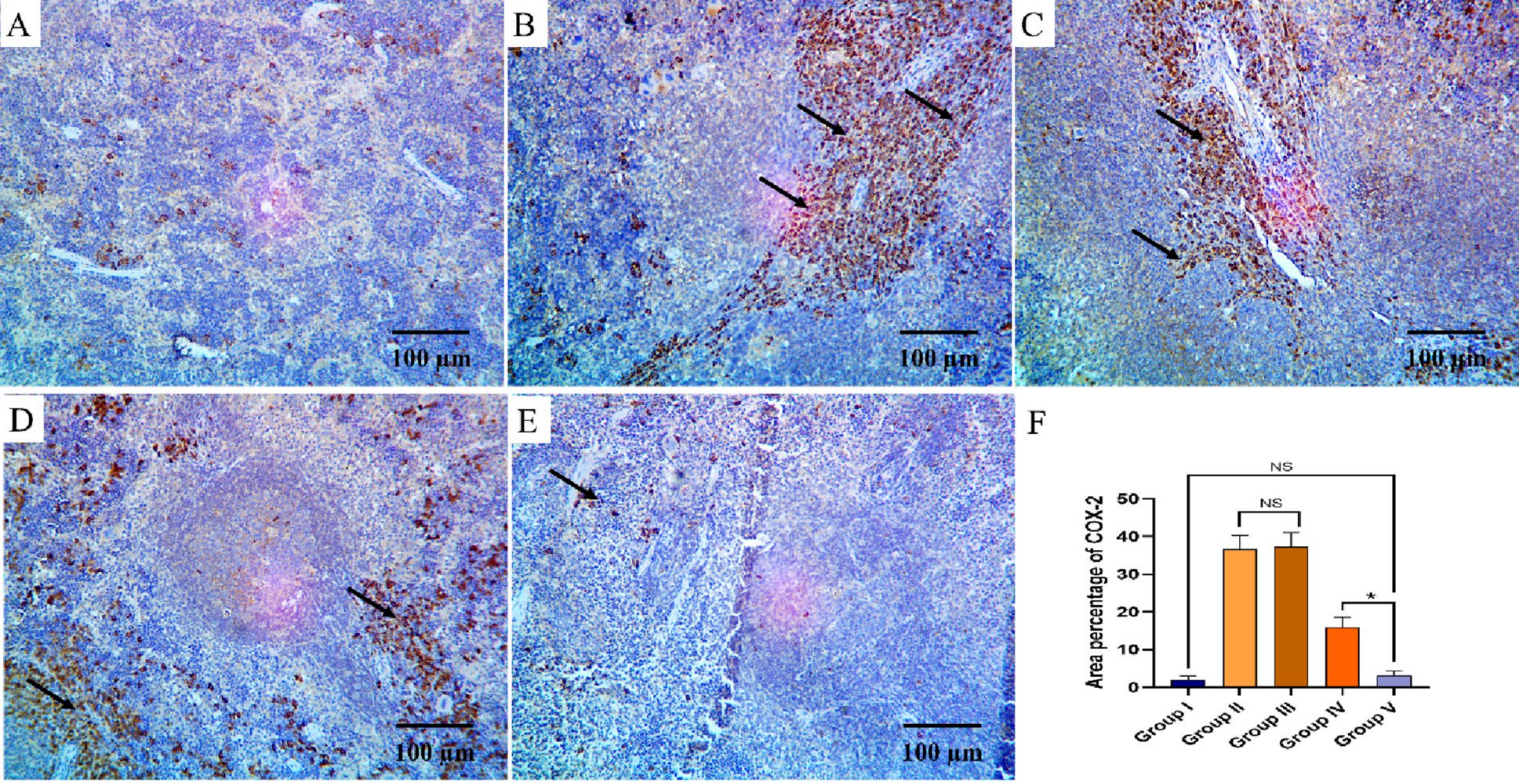
COX-2 immuno-histochemical staining analysis of splenic section in various studied groups: **A** The normal control group express a negative COX-2 reaction. **B**, **C** The positive control and blank groups express strong positive COX-2 reaction in the form of brown stain within the cytoplasm (arrows) with a significant difference with both control group and the PEGylated PLGA NCs loaded with the *P. oxalicum* extract group. **D** The *P. oxalicum* extract group expresses a moderate COX-2 reaction. **E** The PEGylated PLGA NCs loaded with the *P. oxalicum* extract group express few COX-2 positive cells (arrow) and a negative reaction in most of cells with a non-significant difference with the normal control group. **F** Area percentage of COX-2 immune reaction in all groups. The single asterisk denotes a significant (*p* < 0.05) difference, and the abbreviation (NS) denotes a non-significant (*p* > 0.05) difference (COX-2 × 400, scale bar = 50 μm)

The TNF-α and COX-2 immunostaining of the liver, kidney, and spleen of the different groups are shown in Figs. 8, 9, 10, 11, 12 and 13.

### Molecular Docking

Performing molecular docking studies, the binding mechanisms and affinity for the active components on three proteins linked to *C. albicans* were examined. The docking score revealed that the tested ligand had varying binding affinities to the target proteins being studied (Table 5).

**Table 5:** Docking affinities (*Kcal/mol*) for the studied compounds into proteins of *C. albicans*

Protein/Compounds	SAP4	SAP5	SAP6
LA	− 6.8	− 5.5	− 4.4
SA	− 6.4	− 5.9	− 5.5
AME	− 6.2	− 7.6	− 6.0
EA	− 5.8	− 7.7	− 6.1
KA	− 3.9	− 7.1	− 6.5
Pepstatin	− 5.5	− 8.6	− 6.2

**Fig. 14:**
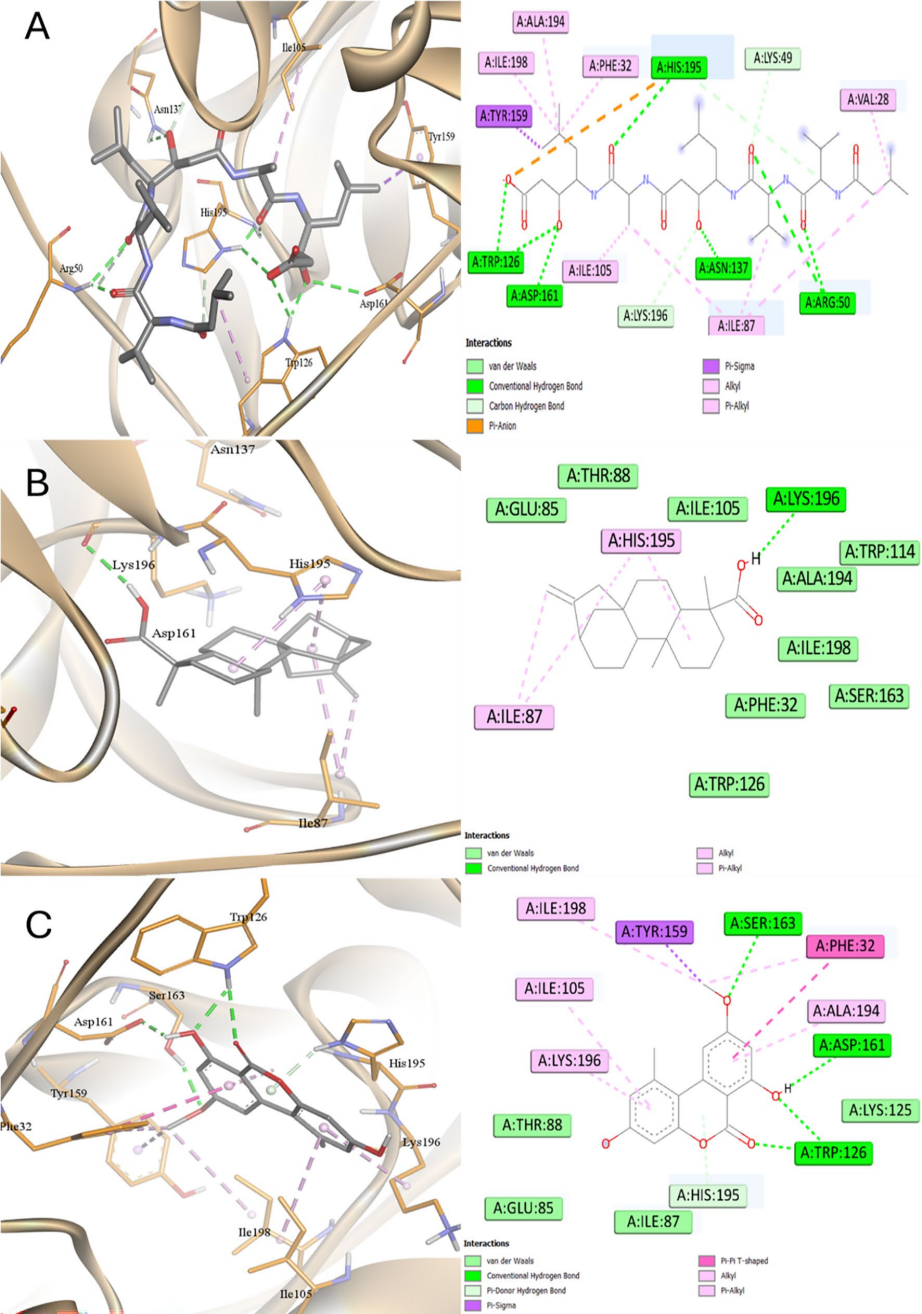
Docking into SAP4 proteins of *C. albicans* **A** Pepstatin, **B** Kaurenic acid, and **C** Alternariol monomethyl ether

**Fig. 15:**
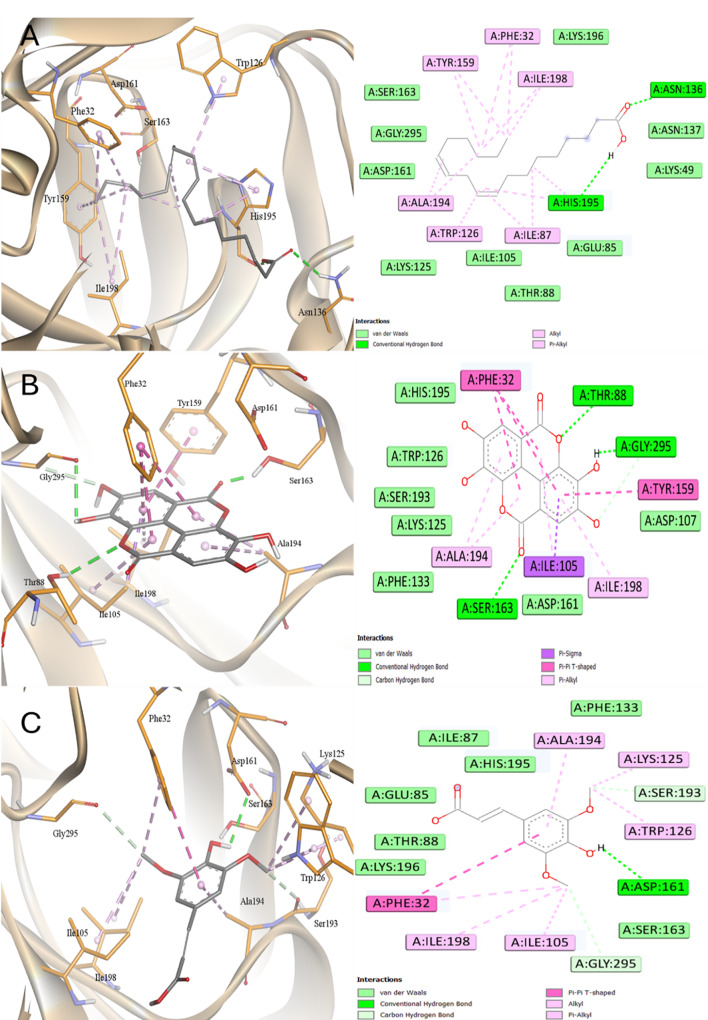
Docking into SAP4 proteins of *C. albicans* **A** Linoleic acid, **B** Ellagic acid, and **C** Sinapinic acid

According to the docking affinities inside SAP4, all ligands accommodated the same active site sharing common interactions in between with His195, Lys196, Asp191, and Tyr159 (Figs. 14 and 15). All ligands showed docking scores greater than Pepstatin (except KA was the worst binding affinity) with LA was the best. KA showed only one H-bond and two hydrophobic interactions while two H-bonds and many hydrophobic interactions were observed with LA. LA interacted with 7 amino acids via hydrophobic interaction while KA interacted with two amino acids via hydrophobic interaction. The order of affinity to SAP4 enzyme was LA > SA > AME > EA > Pepstatin > KA.

**Fig. 16:**
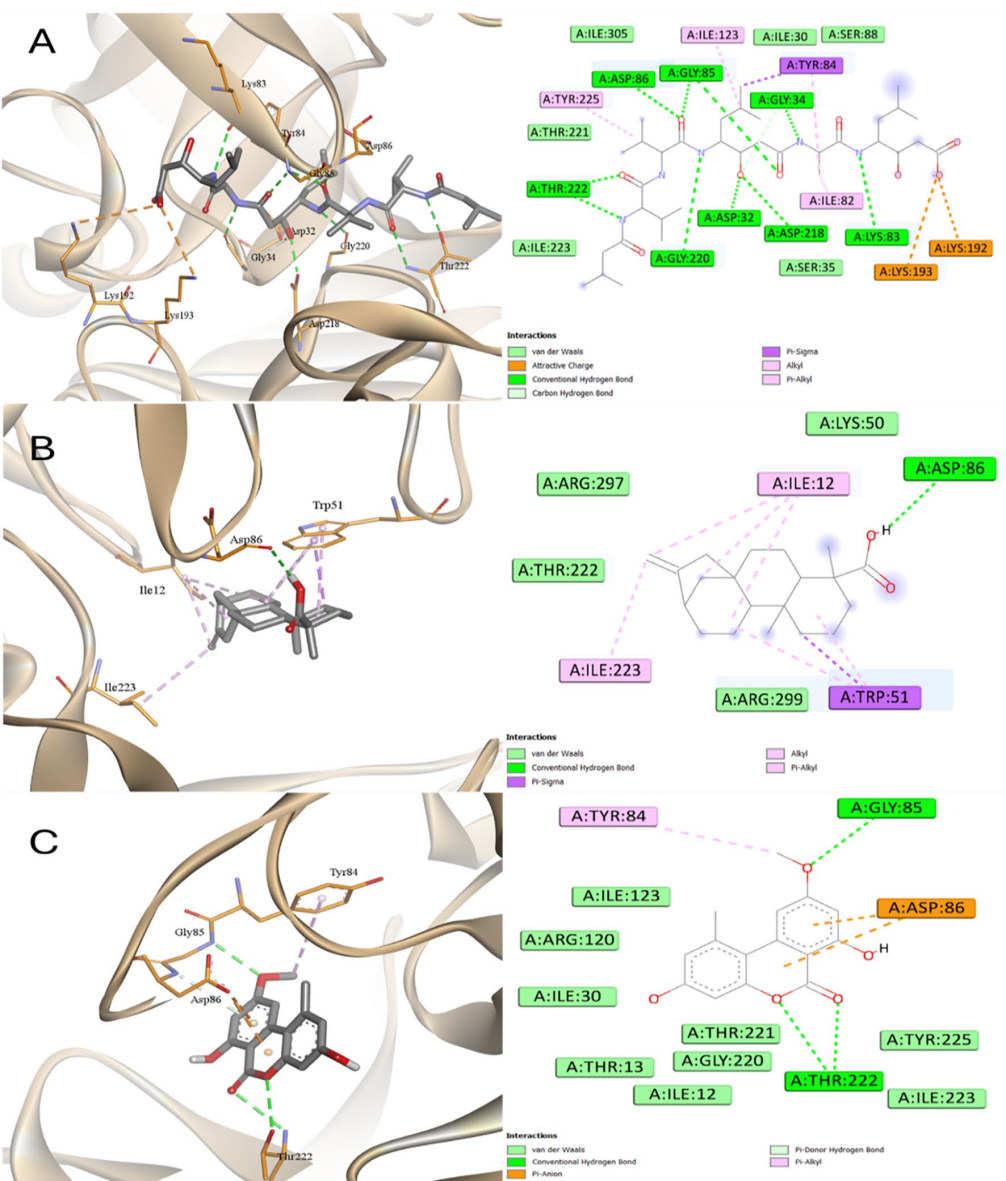
Docking into SAP5 proteins of *C. albicans* **A** Pepstatin, **B** Kaurenic acid, and **C** Alternariol monomethyl ether

**Fig. 17:**
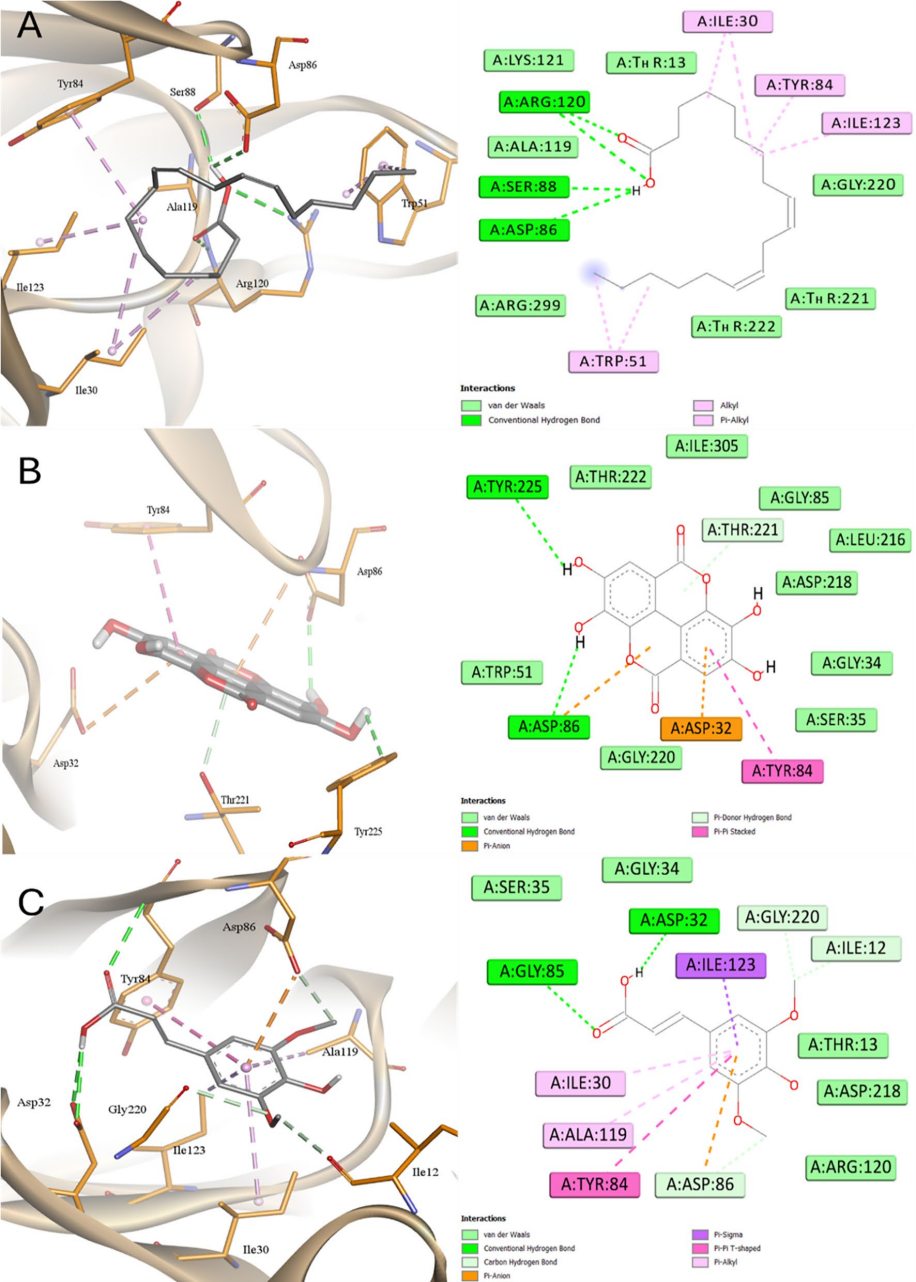
Docking into SAP5 proteins of *C. albicans* **A** Linoleic acid, **B** Ellagic acid, and **C** Sinapinic acid

Regarding the SAP5 target, all ligands exhibited lower affinity than Pepstatin with AME and EA showed the best binding affinities and LA was the worst. Figures 16 and 17 demonstrated binding of all ligands into the same active site of SAP5. AME and EA exhibited a shared interaction with Asp86 and Tyr85. AME demonstrated a hydrophobic interaction with Tyr84, whereas EA engaged in pi-pi interactions with Tyr84, and both compounds interacted through Pi-anion with Asp86. The order of affinity with SAP5 was Pepstatin > EA ~ AME > KA > SA > LA.

**Fig. 18:**
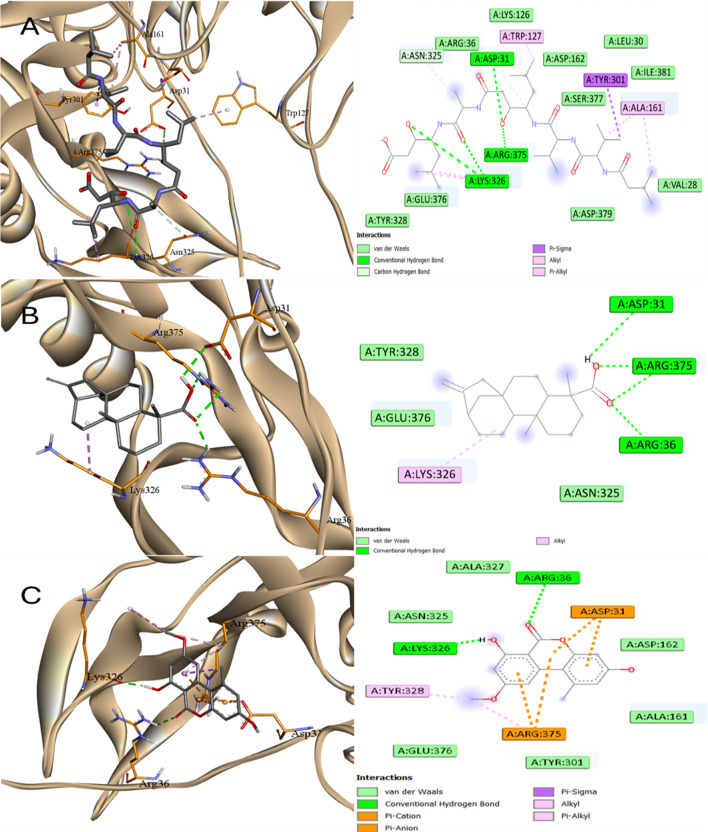
Docking into SAP6 proteins of *C. albicans* **A** Pepstatin, **B** Kaurenic acid, and **C** Alternariol monomethyl ether

**Fig. 19:**
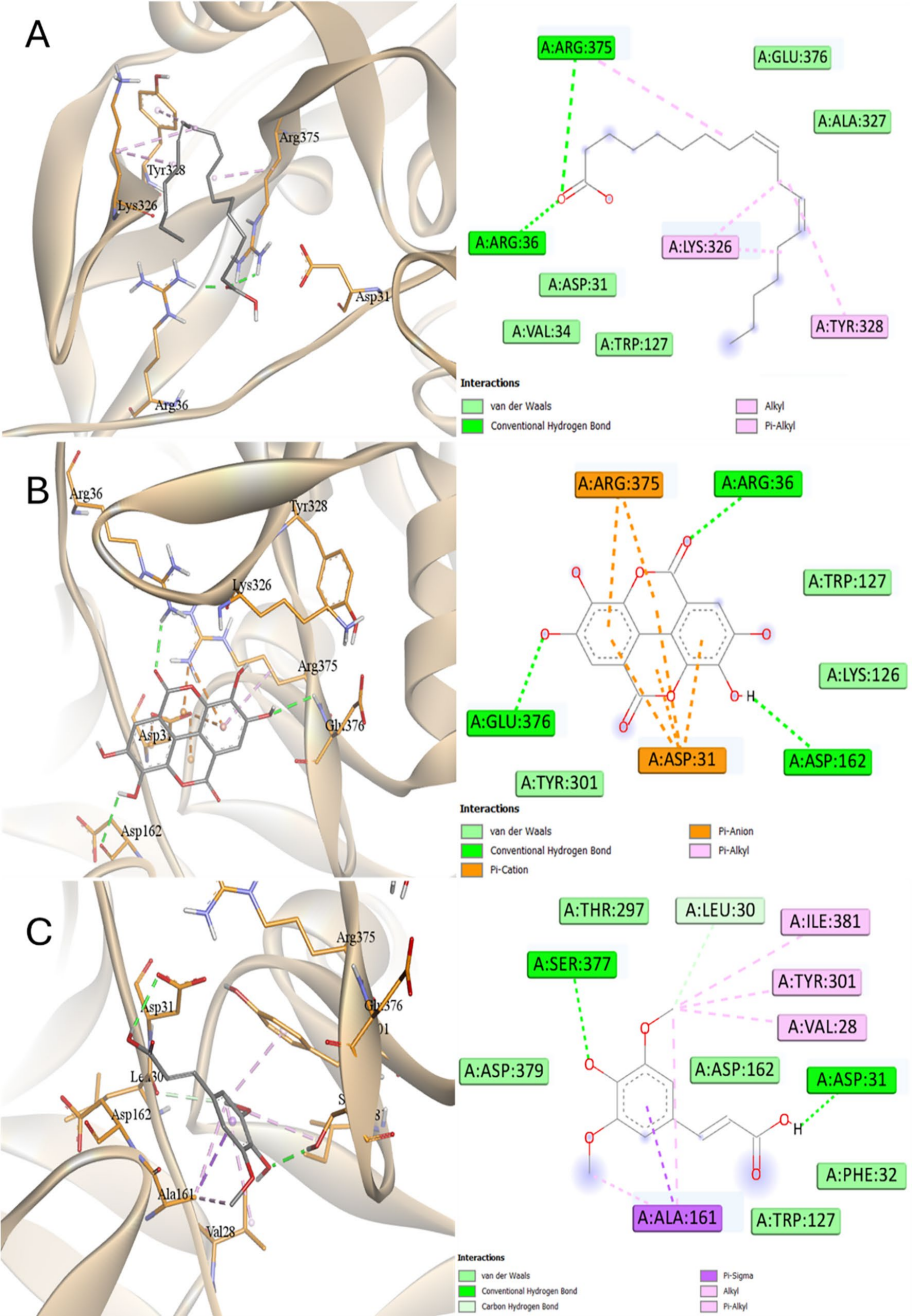
Docking into SAP6 proteins of *C. albicans* **A** Linoleic acid, **B** Ellagic acid, and **C** Sinapinic acid

Inside the active site of SAP6, all compounds showed interactions with Arg375 and Asp31 except LA and SA that showed only one of these two interactions (Figs. 18 and 19). This was reflected to the affinities as all showed similar affinity greater than LA and SA with the order of binding affinities were KA > Pepstatin > EA > AME > SA > LA. KA engaged with three amino acids through hydrophobic interactions, while it was influenced by pi-sigma interactions with Trp51 and hydrogen bonding with Asp86.

## Discussion

The occurrence of black fungal infection during the COVID-19 pandemic has prompted the elucidation of novel antifungal drugs. Natural agents are important alternative sources for antifungal drugs. Endophytic fungi can reside within their host plants; thus, they offer an exclusive bioresource for innovative chemicals that possess a wide array of bioactivities.

*Acalypha hispida* (family Euphorbiaceae) was selected for this study due to its well-documented ethnomedicinal properties, including antimicrobial, anti-inflammatory, and antioxidant activities, which suggest that its associated endophytes might harbour similar or complementary bioactivities [28]. Members of the genus *Acalypha* are also known to contain diverse secondary metabolites such as flavonoids, terpenoids, and phenolic acids that can influence or stimulate metabolite biosynthesis in their endophytic partners [29, 30]. Therefore, investigating the endophytes from *A. hispida* provides an opportunity to uncover novel bioactive compounds with potential antifungal efficacy.

Among the isolated endophytes, *Penicillium oxalicum* was identified based on 16 S rRNA gene sequencing. *P. oxalicum* has been extensively reported as a productive manufacturer of various bioactive metabolites, including polyketides, alkaloids, and terpenoids with antimicrobial and cytotoxic properties [31]. The metabolic versatility of this species and its frequent occurrence as an endophyte make it a valuable target for natural product discovery. The ability of *P. oxalicum* to produce diverse bioactive compounds is also linked to its genetic richness, containing multiple biosynthetic gene clusters encoding nonribosomal peptide synthetases (NRPS) and polyketide synthases (PKS), which contribute to the generation of structurally diverse metabolites [31].

Metabolite analysis using UHPLC-MS/MS showed that the identified metabolites produced by the endophytic extract, *P. oxalicum*, are classified into various phytochemical categories, including long-chain fatty acids, coumarins, diterpenoids, polyketides, eudesmane, isoeudesmane, and cycloeudesmane sesquiterpenoids, which is consistent with previous studies [32–35]. Our study revealed that linoleic acid is the most abundant metabolite produced by the extract of *P. oxalicum*. This result was in agreement with the previous work in which linoleic acid was converted by the enzyme 8R,11 S-linoleate diol synthase (8R,11 S-LDS) from *P. oxalicum* into its metabolites: mono-hydroxy linoleic acid (8R-HODE) and dihydroxy linoleic acid (7S,8S-DiHODE) [36]. All the above evidence has shown that *P. oxalicum* can use different enzymatic routes for the production of a range of metabolites from linoleic acid [37]. Moreover, linoleic acid is also known to exhibit antibacterial properties [38]. Furthermore, some metabolites reported by our study, kaurenic acid, alternariol monomethyl ether, sinapinic acid, and ellagic acid, have been formerly reported to display antimicrobial action [39–43].

Collectively, these findings demonstrate that the *A. hispida* and *P. oxalicum* endophytic association represents a promising biological system for the exploration of novel natural products. The combination of a bioactive host plant and a metabolically versatile endophyte provides a rational and scientifically justified approach to discovering new antifungal agents that could help us in our battle against the emerging drug resistance problem.

The observed antifungal efficacy in this study can be attributed to the combined effects of several bioactive metabolites, each contributing through different mechanisms. Ilicic acid (ilicicolin H) is a polyketide known to inhibit fungal respiratory enzymes, thereby impairing energy metabolism and reducing growth [44]. Sinapic acid exerts antifungal effects primarily through oxidative stress induction and disruption of fungal membrane integrity [45]. Norlichexanthone, a xanthone derivative, has demonstrated inhibition of fungal spore germination and biofilm formation, enhancing overall fungistatic activity [46]. Ellagic acid contributes antioxidant properties while simultaneously exerting direct antifungal activity by interacting with fungal cell walls and membranes, thereby increasing susceptibility to other metabolites [47]. Kaurenic acid, a diterpenoid, shows fungicidal potential against dermatophytes and plant-pathogenic fungi by disrupting cell membrane architecture [48]. 2-Phenylethyl β-d-glucopyranoside, a phenylethanoid glycoside, exhibits activity against *Candida* species, likely through interference with membrane function and yeast-to-hyphae transition [49]. Finally, Petroselinic acid, a monounsaturated fatty acid, has been shown to inhibit hyphal formation, biofilm development, and growth of *Candida albicans*, acting on key metabolic enzymes [50].

The incorporation of these metabolites into nanocapsule formulations significantly enhances their bioavailability, protects labile compounds from enzymatic degradation, and facilitates targeted delivery to fungal-infected tissues. The nanocapsules improve penetration into fungal cells and infected tissue sites, ensuring sustained release and synergistic interaction among metabolites. This method of drug delivery not only amplifies the antifungal potency of the extract but also reduces its potential cytotoxicity via controlling localized concentrations.

Our formulation strategy aims to encapsulate the endophytic fungal extract of *P. oxalicum* via a combined single o/w emulsion /nanoprecipitation method. During the preparation of NCs loaded with the *P. oxalicum* extract, we utilized a mixture of aqueous miscible/partially miscible/non-miscible organic solvents (acetone, DMF, and DCM) to confirm the ultimate solubility of the whole content of the *P. oxalicum* extract and overcome the great diversity in its physicochemical properties.

The basic step of both single emulsion and nanoprecipitation techniques is emulsion formation. Secondly, the polymeric NCs would be fabricated, and herein is the difference between the single emulsion and nanoprecipitation methods. NCs are formed by solvent shifting in nanoprecipitation or by solvent evaporation in a single emulsion [51, 52]. The scientific concept of the nanoprecipitation technique is based on the theory of interfacial disposition. As the polymer desolvation occurs upon its movement from a solvent to a non-solvent phase [53]. In a single emulsion solvent evaporation technique, the polymeric solution would be emulsified in the external aqueous phase, followed by the evaporation of the solvent, resulting in the precipitation of the polymer encapsulating the active candidates [54, 55]. Both techniques are easily applied, cost-effective, and reproducible with a simple scaling-up [56].

Therefore, the NCs are prepared not purely by emulsion–solvent evaporation nor by classic nanoprecipitation, but rather through a synergistic process: emulsification to disperse the oily phase into nanoscale droplets, and nanoprecipitation during solvent removal to stabilize the polymer shell. This hybridization improves entrapment efficiency, stability of *P. oxalicum*, and allows finer control of particle size compared to either method alone.

The greater viscosity on the internal organic phase produced at a higher m-PEG-PLGA concentration results in heavier consistency with larger oily globules not easily broken at the same sonication speed used for formulae prepared with a lower m-PEG-PLGA concentration [56]. This finding clarifies the rationale for the observed increase in NC particle size with higher concentrations of mPEG-PLGA.

In our study, the measurement recorded by the zetasizer showed a higher value compared to the detected diameter of NCs examined under the TEM. This could be attributed to the different mechanisms used in both techniques. Zetasizer depends mainly on the dynamic light scattering (DLS) behavior of the nanosystem that determines the size through the hydrodynamic pattern of NCs, whereas TEM focuses on the visualization of the actual photo of NCs under the microscope [57].

The same method of preparation has been employed in all formulae, and the processing variables, like the stirring rate and the sonication speed, have been kept unchanged. Only one formulation variable has been changed (the concentration of m-PEG-PLGA). At higher polymer concentrations, the organic phase viscosity increased, and the polymer became more available at the oil–water interface, both of which stabilized the emulsion droplets and minimized coalescence. Additionally, the presence of more polymer accelerated shell precipitation during solvent removal, yielding NCs of a more uniform size distribution. This explains the observed decrease in PDI with increasing polymer concentration, in line with reports that enhanced interfacial stabilization and reduced Ostwald ripening improve NCs’ size homogeneity [58].

The negative values of the zeta potential increased at higher m-PEG-PLGA concentrations could be explained by the carboxylic acid groups of glycolic and lactic acids. These carboxylic acid groups have been associated with higher m-PEG-PLGA concentrations, leading to a condenser negative charge covering the surface of NCs [58].

The prepared PEGylated PLGA NCs loaded with the *P. oxalicum* extract were tested in vitro and in vivo to assess their antifungal efficacy on *C. albicans* isolates. Interestingly, a promising antifungal action was revealed by the prepared formulation with enhanced in vivo efficacy, which was comparable to the normal control group. Regarding the in-silico studies, good affinities were obtained with the three target enzymes of *C. albicans*, with LA being the highest towards SAP4, while EA and AME were the highest towards SAP5 and KA towards SAP6, suggesting the possible mechanisms for their activity on *C. albicans*.

Regarding the antifungal activity by comparing free *P. oxalicum* extract to PEGylated PLGA NCs, it has been found that PEGylated PLGA NCs have a significant enhancement with an appearance similar to the healthy architecture of liver, kidney, and spleen tissues as visualized by H& E stain (Figs. 5, 6 and 7), and also confirmed by TNF-α (Figs. 8, 9 and 10) and COX-2 (Figs. 11, 12 and 13) immuno-histochemical staining. This effect likely derives from a combination of improved pharmacokinetics, biodistribution, and expected sustained release: PEGylation confers “stealth” behavior by reducing opsonization and clearance by the mononuclear phagocyte system, thereby prolonging systemic circulation and increasing the likelihood of accumulation in infected organs [59]. The common initial burst of PLGA matrix assures a higher local concentration of the plant extract in diseased tissues, likely accelerating fungal clearance and suppressing inflammation, enabling tissue repair and structural recovery [60]. Importantly, the biocompatibility of PEG-PLGA minimizes carrier-induced toxicity, so the observed restoration reflects pharmacological, not merely structural, benefits. These findings are consistent with prior reports that PEGylated PLGA systems can reduce reticuloendothelial uptake and prolong half-life, thereby improving therapeutic index in systemic delivery [61].

Although the PEGylated PLGA nanocapsules loaded with *P. oxalicum* extract demonstrated significant antifungal activity in vitro, in vivo, and in silico, several aspects require further investigation. Future studies should evaluate the long-term stability of the NCs under different storage conditions, assess the feasibility of large-scale production, and conduct comprehensive in vivo safety assessments, including biodistribution, immunogenicity, and potential off-target effects. Addressing these factors will be essential to confirm the safety and efficacy of this novel antifungal formulation and to support its potential translation toward clinical applications.

## Conclusion

The prepared PEGylated PLGA NCs loaded with the *P. oxalicum* extract were tested in vitro and in vivo to assess their antifungal efficacy on *C. albicans* isolates. Interestingly, a promising antifungal action was revealed by the prepared formulation with enhanced in vivo efficacy, which was comparable to the normal control group. Regarding the in-silico studies, good affinities were obtained with the three target enzymes of *C. albicans*, with LA being the highest towards SAP4, while EA and AME were the highest towards SAP5 and KA towards SAP6, suggesting the possible mechanisms for their activity on *C. albicans*.

## Supplementary Information

Below is the link to the electronic supplementary material.

Supplementary Material 1.

## Data Availability

No datasets were generated or analysed during the current study.
