# Supplementary material for: Antifungal activity of oily core PEGylated PLGA nanocapsules loaded with Penicillium oxalicum fungal extract: in vitro, in vivo, and in silico study
Source: Microb Cell Fact. 2026 Jan 16;25:37. doi: 10.1186/s12934-025-02891-x (PMC12874840; doi:10.1186/s12934-025-02891-x)
Supplement: Supplementary file 1 — Supplementary Material 1. [file 12934_2025_2891_MOESM1_ESM.docx]

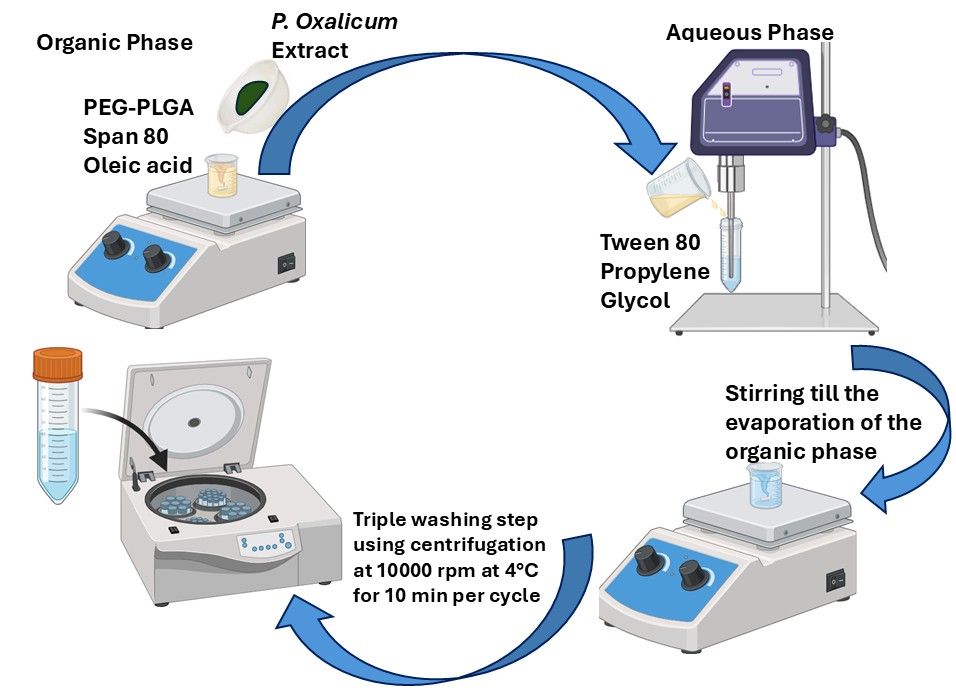


**Figure S1.** Schematic presentation showing the preparation method of PEGylated PLGA NCs loaded with *P. oxalicum.*


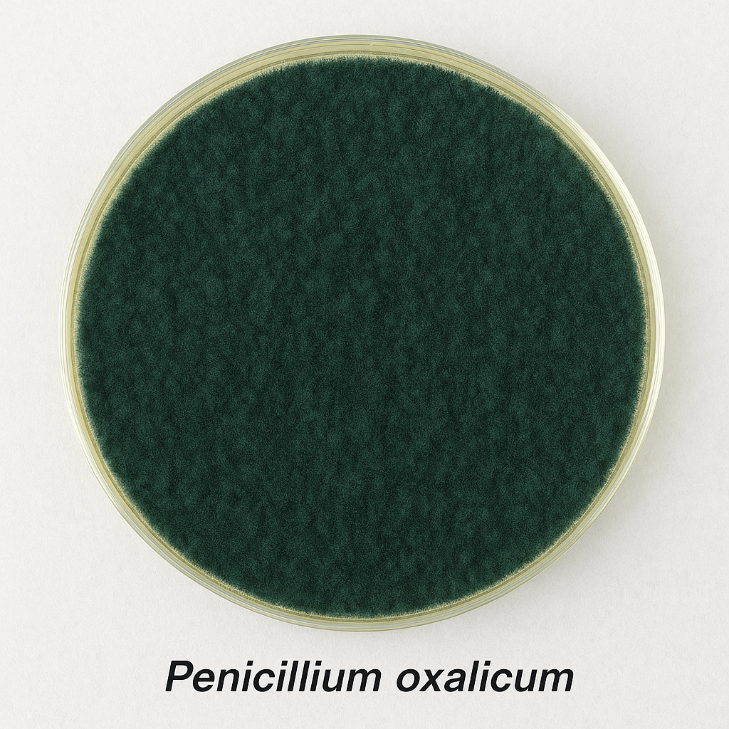


**Figure S2.** Colony morphology of *Penicillium oxalicum* isolated from the leaves of *Acalypha hispida* on PDA medium.


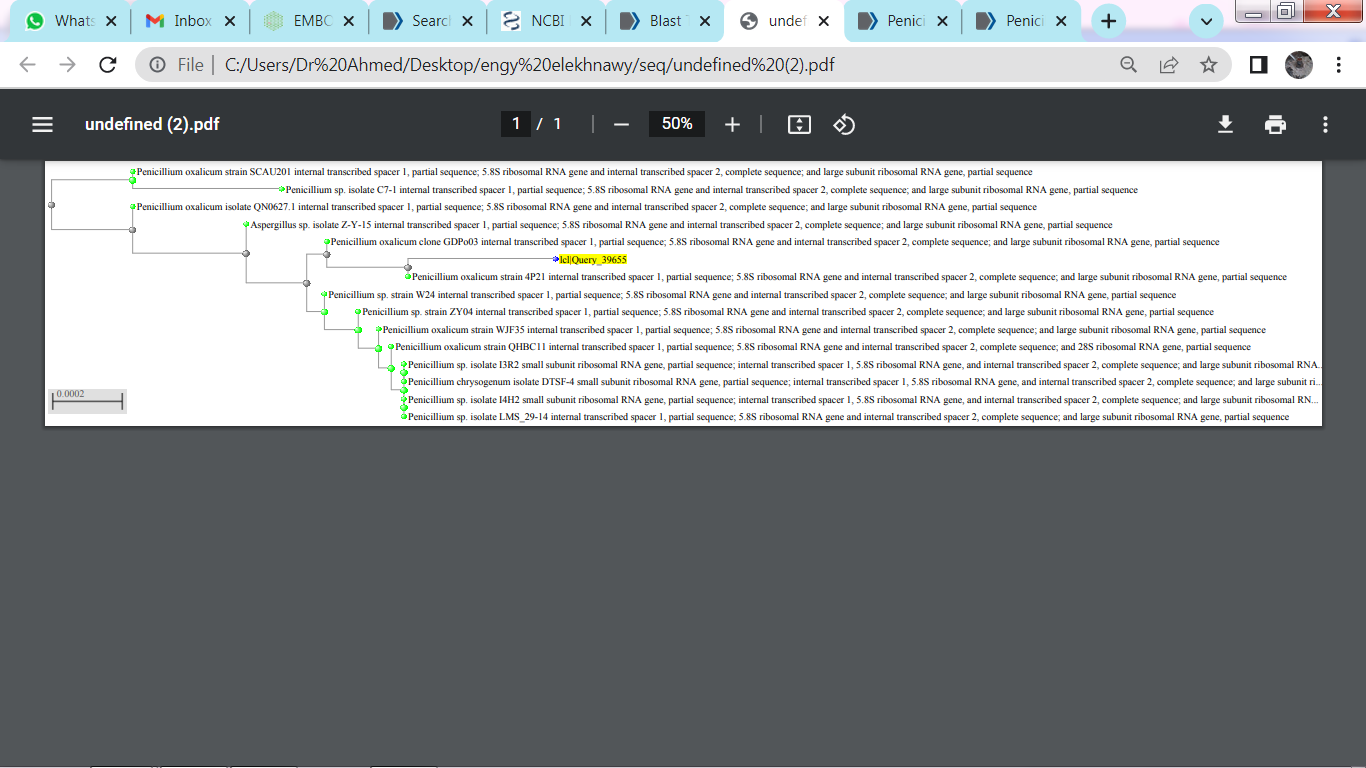


**Figure S3.** The phylogenetic tree based on the 18S rRNA gene sequence of the isolated endophytic fungus, *P. oxalicum* is indicated by the yellow highlight.


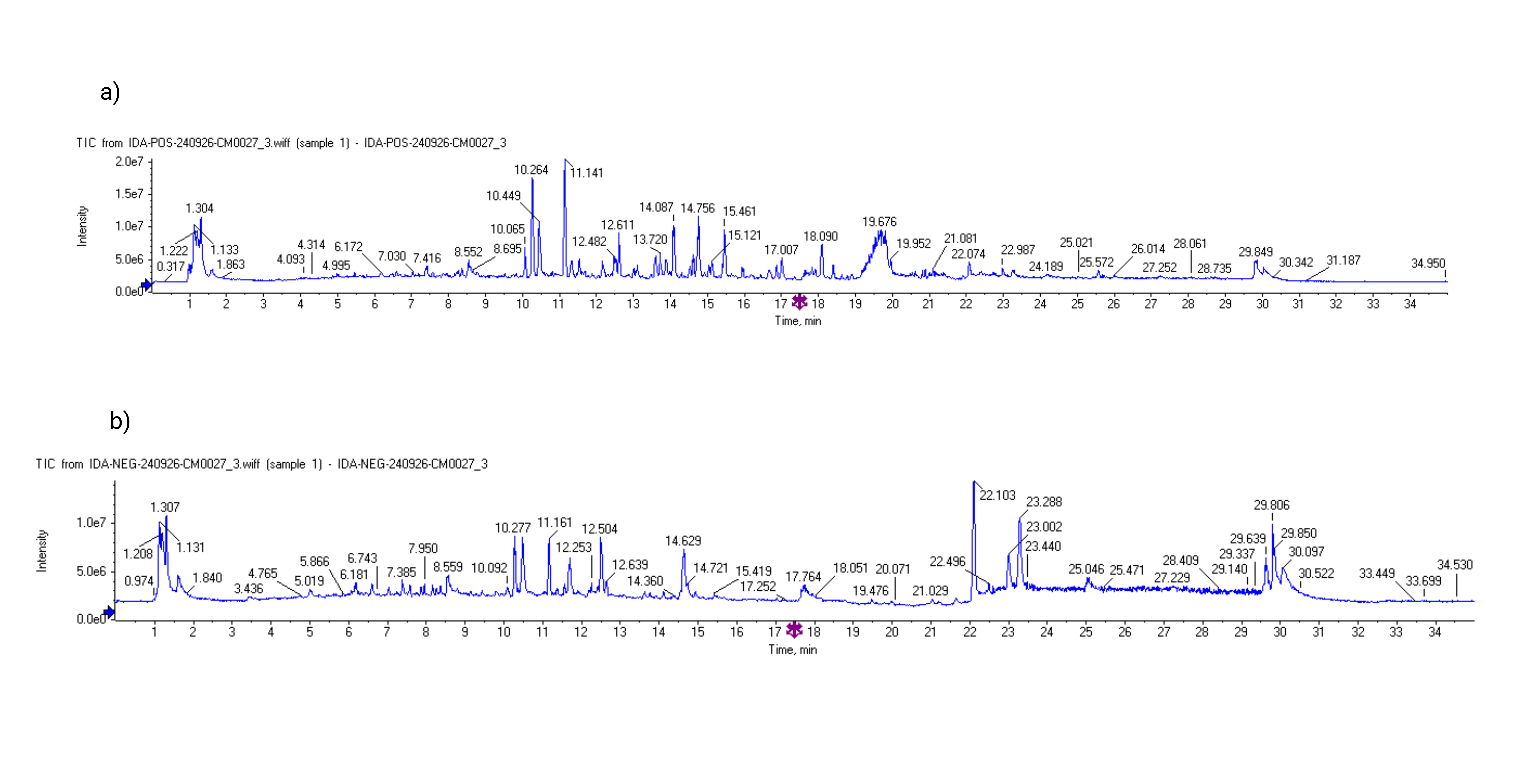


**Figure S4.** The total ion chromatography (TIC) of the extract of *Penicillium oxalicum* in both a) positive, and b) negative modes.


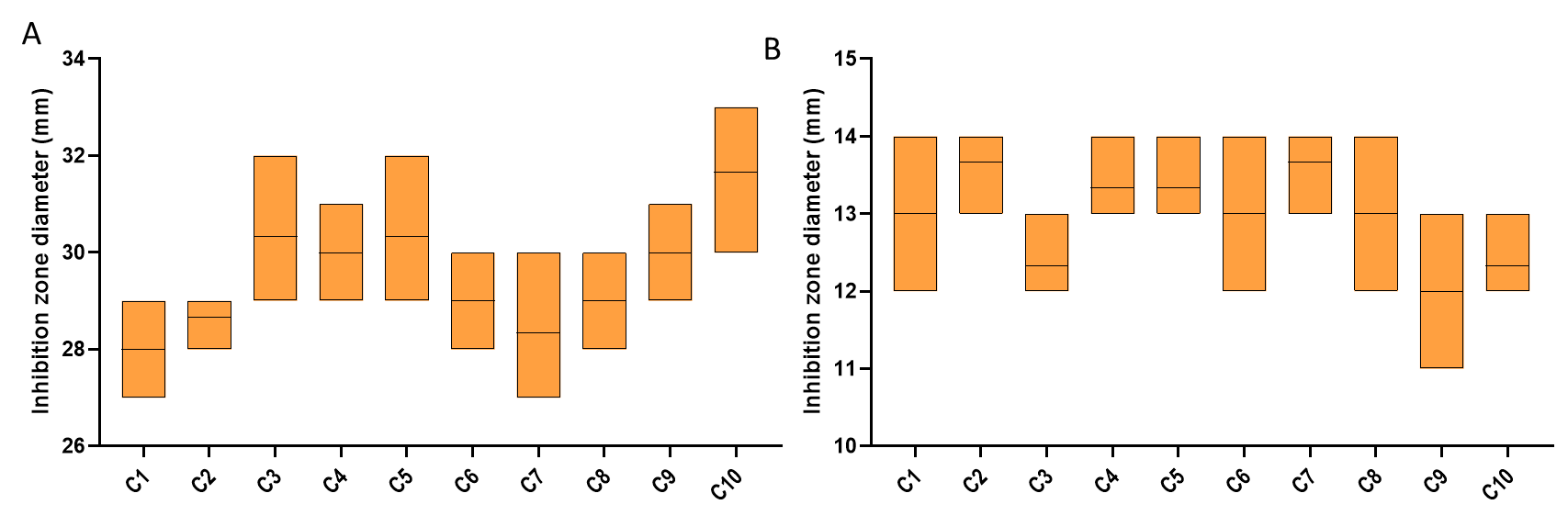


**Figure S5.** Inhibition zone diameters of: A) the fungal extract and B) the PEGylated PLGA NCs loaded with the *P. oxalicum* extract. There was a significant increase (*p*<0.05) in the diameters of the inhibition zones of the PEGylated PLGA NCs loaded with the *P. oxalicum* extract in relation to the fungal extract, which means a significant increase in the antifungal action.


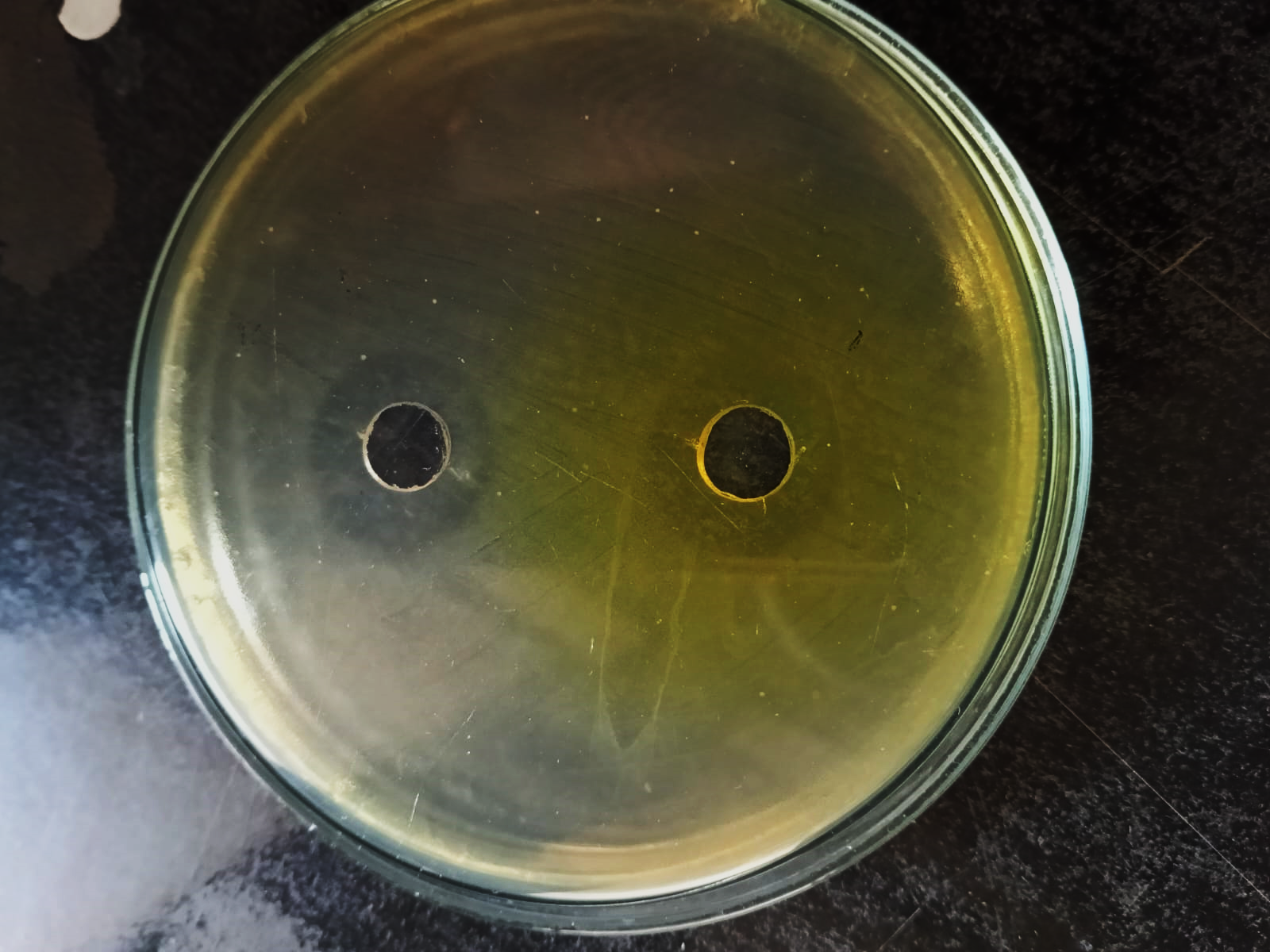


**Figure S6.** A representative example for agar diffusion test result of the fungal extract.


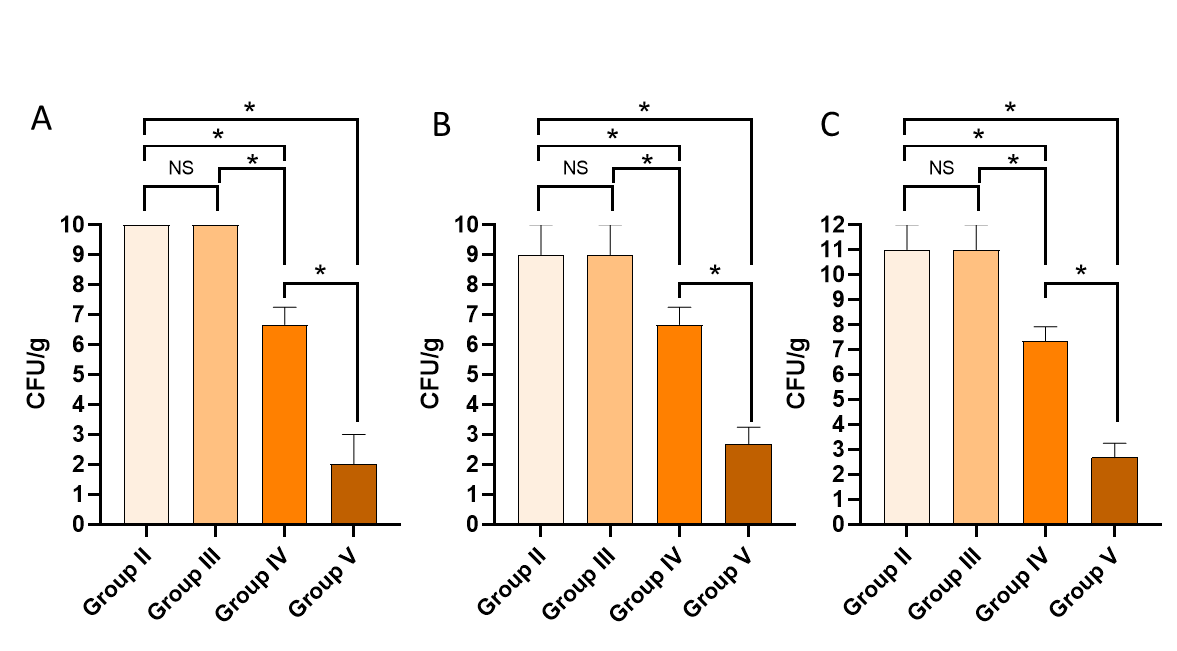


**Figure S7.** Fungal burden in the different groups. The single asterisk represents a significant difference (*p*<0.05) and the abbreviation (NS) means a non-significant difference (*p*<0.05).
